# Associations of preconception retinal microvasculature with lipid profiles from mid-pregnancy to three months postpartum

**DOI:** 10.1038/s44325-026-00130-9

**Published:** 2026-05-15

**Authors:** Mengjiao Liu, Jiayi Shen, Johan G. Eriksson, Yap Seng Chong, Jerry Chan, Andrea Leiva, Sebastian E. Illanes, Ling-Jun Li

**Affiliations:** 1 School of Public Health, Jiangxi Medical College, Nanchang University, Jiangxi, China; 2 Jiangxi Provincial Key Laboratory of Preventive Medicine, Nanchang University, Jiangxi, China; 3 Department of Obstetrics & Gynaecology, Yong Loo Lin School of Medicine, National University of Singapore, Singapore, Singapore; 4 Human Potential Translational Research Programme, Yong Loo Lin School of Medicine, National University of Singapore, Singapore, Singapore; 5 Department of General Practice and Primary Health Care, University of Helsinki, Helsinki, Finland; 6 Folkhälsan Research Center, Helsinki, Finland; 7 Duke-NUS Medical School, Singapore, Singapore; 8 KK Women’s and Children’s Hospital, Singapore, Singapore; 9 Faculty of Medicine and Science, Universidad San Sebastian, Santiago, Chile; 10 Reproductive Biology Program, Center for Biomedical Research and Innovation, Universidad de los Andes, Santiago, Chile; 11 IMPACT, Center of Interventional Medicine for Precision and Advanced Cellular Therapy, Santiago, Chile; 12 Faculty of Medicine, School of Medicine, Universidad de Los Andes, Santiago, Chile; 13 Department of Obstetrics & Gynaecology, Li Ka-Shing Faculty of Medicine, The University of Hong Kong, Hong Kong SAR, China

**Keywords:** Endocrine system and metabolic diseases, Cardiovascular diseases

## Abstract

We examined associations between preconception retinal microvasculature and maternal dyslipidemia at mid-pregnancy and three months postpartum in 366 women (mean age 29.9 years). Preconception retinal vascular morphology assessed arteriolar and venular caliber, fractal dimension, and branching angle. Lipids were measured at 24–28 weeks’ gestation and at three months postpartum. We analyzed relationships with each time point separately, overall change, and classification by clinical cut-offs for suboptimal lipid status using 187 complete data. Wider venular caliber, higher arteriolar fractal dimension, and larger arteriolar branching angle were associated with adverse lipid changes; other retinal parameters showed no association. Notably, each standard deviation increase in arteriolar branching angle was linked to higher odds of persistently suboptimal total cholesterol (OR 2.23, 95% CI 1.25, 4.00) and LDL cholesterol (OR 3.20, 95% CI 1.46, 6.85) from mid-pregnancy to three months postpartum. Preconception retinal imaging may add value to antenatal and postpartum maternal dyslipidemia risk classifications and future cardiovascular disease.

## Introduction

Women undergo substantial changes in lipid metabolism during pregnancy due to increased hepatic lipid synthesis and reduced activity of lipoprotein lipase^1^. In normal pregnancies, the total cholesterol (TC), high- and low-density lipoprotein cholesterol (HDL-C & LDL-C) levels increased by approximately 25 ~ 50%, as well as triglycerides (TG) increased by 2–3 fold since 8–12 weeks of gestation^1,2^. Such an increment of maternal lipids is essential for fetal growth and development. However, if such an increment exceeds the biological demand, pregnancy could be compromised by dyslipidemia, with an extreme condition called maternal supraphysiological hypercholesterolemia (MSPHC)^3^.

Evidence showed that elevated supraphysiological levels of lipids during pregnancy were associated with maternal oxidative stress, damaged vascular endothelium, and the endothelial dysfunction of the placental microvasculature^3^. Thus, MSPHC could also lead to several adverse outcomes during pregnancy, such as pre-eclampsia and gestational diabetes mellitus (GDM), as well as intrauterine growth restriction and preterm birth in their offspring^4^. This evidence suggests that gestational dyslipidemia could be an important contributor to a long-term cholesterol burden of women after pregnancy, which could lead to long-term cardiovascular disease (CVD) risk in women, such as a higher risk of hypertension and metabolic syndrome^5,6^. It has been suggested that the ideal time to identify women’s lipid profile is before conception^3^, and the European Heart Association suggested incorporating lipid testing into the routine prenatal checkup^7^. Since lipid testing is not routinely performed before conception and instead relies heavily on patient-initiated preconception counseling, this may limit effective preventive management for women of childbearing age at risk of antenatal dyslipidemia. At the same time, there are few reliable methods to evaluate whether a woman will develop dyslipidemia during pregnancy. Identifying specific high-risk groups could provide valuable insights into the health outcomes of mothers and their offspring.

Retinal imaging is an advanced technology that can be used to study general microcirculation non-invasively^8^. In the general population, abnormal retinal vascular morphology, such as retinal arteriolar narrowing and venular widening, is associated with a higher risk of dyslipidemia, obesity, and CVD^9,10^. Our research team has further demonstrated that retinal microvascular parameters measured during mid-to-late pregnancy are associated with maternal antenatal conditions, including gestational hypertension and GDM, as well as longer-term cardiometabolic outcomes such as metabolic syndrome and glucose abnormalities up to five years postpartum^11,12^. These findings underscore a robust biological link between retinal microvascular changes and systemic metabolic regulation during pregnancy. With the integration of artificial intelligence, retinal imaging can now deliver results efficiently, supporting its growing utility in clinical and population-based research^13^. This technological advancement opens new avenues for investigating early metabolic disturbances in pregnancy and may, in the future, contribute to a more nuanced understanding of gestational dyslipidemia risk.

In order to find out whether maternal retinal microvasculature during the preconception phase could provide valuable information about the course of suboptimal lipid profiles during pregnancy and early postpartum, we conducted an exploratory longitudinal analysis to assess the association between preconception maternal retinal microvasculature and maternal lipid profiles from mid-late pregnancy to three months postpartum. We hypothesized that abnormal retinal microvascular features, such as wider retinal venular caliber or greater retinal branching angles during the preconception phase, could be associated with an increased risk of suboptimal lipid profile during pregnancy and even postpartum.

## Results

**Fig. 1:**
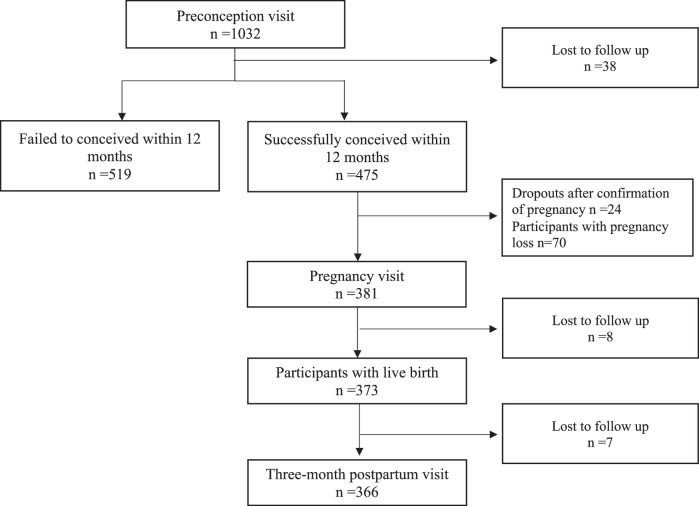
Participant flowchart. The participants enrolled in the Singapore PREconception Study of long-Term maternal and child Outcomes (SPRESTO) cohort and those analyzed in the current study.

A total of 1032 eligible women were recruited during the preconception phase, and 475 of them successfully conceived within a 12-month follow-up. 381 women participated in the pregnancy visit, and 373 had live births, most of which (*n* = 366) participated in the three months postpartum visit (Fig. 1). We included women with both preconception retinal examination (*n* = 189) and lipids measured during pregnancy (*n* = 328) and three months postpartum (*n* = 284) in the final analysis (*n* = 187). We compared the included and excluded participants in Supplementary Table 2. Overall, demographic characteristics, retinal vascular parameters, and most lipid measures were similar between the two groups. The only statistically significant difference observed was slightly higher preconception HDL cholesterol among excluded participants compared with included participants (1.50 ± 0.28 vs 1.43 ± 0.28 mmol/L, *p* = 0.04).

The mean age at the pregnancy visit was 29.9 years; 75% were Chinese, and 70.2% had a college degree (Supplementary Table 1). 60.8% of women were nulliparous. Only 7.6% of the women smoked, while 68.9% consumed alcohol before pregnancy. The mean gestational weeks at delivery were 39.2 weeks, and the mean gestational weight gain was 11.2 kg.

Based on the clinical practice guidelines of Singapore, only 13.6% (25/184) of mothers had suboptimal total cholesterol at 24–28 weeks of gestation, and at three months postpartum, 36.1% (56/155) of mothers had suboptimal total cholesterol. Thus, from mid-pregnancy to three months postpartum, 62.6% (97/155) of mothers always had optimal total cholesterol, and 26.4% (41/155) and 11.0% (17/155) of mothers had ever or persistently suboptimal total cholesterol from pregnancy to postpartum (Table 1). Similarly, 66.5% of mothers had optimal LDL-C, and 25.8% and 7.7% of mothers had ever or persistently suboptimal LDL-C from pregnancy to postpartum.

**Table 1:** Number of participants with suboptimal lipid status (*n* = 155 ~ 184)

Suboptimal lipids	Total cholesterol *N* (%)	Low-density lipoprotein cholesterol *N* (%)
24–28 weeks of gestation	(≥ 7.5 mmoI/L)	(≥4.4 mmoI/L)
	25 (13.6)	23 (12.6)
Three months postpartum	(≥5.2 mmol/L)	(≥3.4 mmol/L)
	56 (36.1)	45 (29.0)
Optimal	97 (62.6)	103 (66.5)
Ever suboptimal	41 (26.4)	40 (25.8)
Persistently suboptimal	17 (11.0)	12 (7.7)

Definition of suboptimal was according to the clinical practice guidelines of Singapore

Total cholesterol: <5.2 mmol/L optimal; ≥5.2 mmol/L suboptimal; ≥7.5 mmoI/L during pregnancy

Triglycerides: <2.3 mmol/L optimal; ≥2.3 mmol/L suboptimal;

High density lipoprotein cholesterol: <1.0 mmol/L suboptimal; ≥1.0 mmol/L optimal.

### Preconception retinal microvasculature and maternal lipids measured at mid-pregnancy and three months postpartum

Per one standard deviation (SD) increment of arteriole fractal dimension was associated with lower HDL-C measured during pregnancy (*β* = −0.16 SD, 95% CI −0.31, −0.02); each SD increment of arteriole branching angle was associated with higher total cholesterol (*β* = 0.17 SD, 95% CI 0.01, 0.32) and LDL-C (*β* = 0.19 SD, 95% CI 0.04, 0.35) measured during pregnancy, after adjusting for age, ethnicity, parity, education, pre-pregnancy BMI, preconception smoking status and family history of diabetes (Supplementary Table 3)

**Fig. 2:**
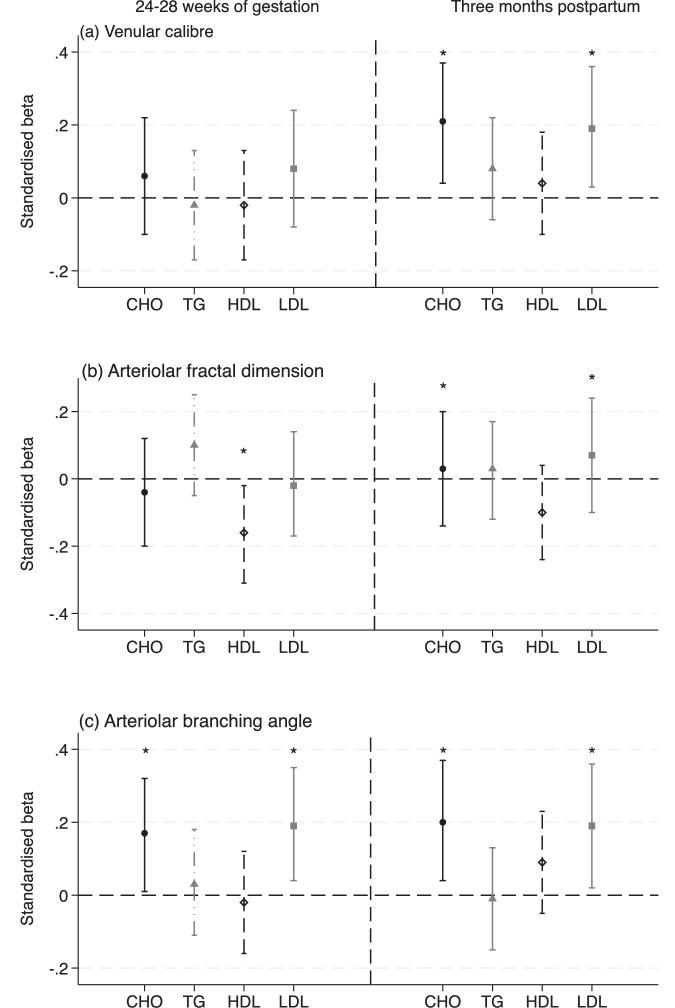
Associations between preconception venular caliber, arteriolar fractal dimension, branching angle and maternal lipids measured during pregnancy (left) and three months postpartum (right). **a** Venular caliber, **b** Arteriolar fractal dimension, **c** Arteriolar branching angle. The circle, triangle, hollow diamond, and squares are beta estimates for total cholesterol, triglycerides, high-density, and low-density lipoprotein cholesterol (per SD change). Vertical bars indicate 95% confidence intervals of estimates. * indicates *p* < 0.05. The regression models were adjusted for age, ethnicity, education, parity, family history of diabetes, pre-pregnancy smoking status and body mass index.

For maternal lipid profiles measured at three months postpartum, we found that one SD increase in retinal venular caliber was positively associated with 0.21 SD (95% CI 0.04, 0.37) total cholesterol and 0.19 SD (95% CI 0.03, 0.36) LDL-C at three months postpartum but not during pregnancy, after adjusting for age, ethnicity, parity, education, pre-pregnancy BMI, smoking stating status and family history of diabetes (Supplementary Table 3). For retinal geometric parameters, the arteriolar branching angle was associated with lipid concentrations, whereas the venules were not (Fig. 2).

### Associations between preconception retinal microvasculature and maternal lipids levels across mid-pregnancy to three months postpartum

Applying a linear mixed model (Table 2), similar to what we found in the separate linear regression models, the arteriole fractal dimension (per SD increase) showed positive associations with triglycerides (0.12 SD, 95% CI 0.01, 0.24) but attenuated after adjusting for potential confounders (0.06 SD, 95% CI −0.05, 0.18); and arteriole fractal dimension (per SD increase) was negatively associated with HDL-C in both unadjusted (*β* = −0.20 SD, 95% CI −0.32, −0.08) and adjusted model (*β* = −0.14 SD, 95% CI −0.25, −0.02). Arteriole branching angle (per SD increase) was positively associated with total cholesterol (*β* = 0.15 SD, 95% CI 0.01, 0.28) and LDL-C (*β* = 0.15 SD, 95% CI 0.02, 0.29) after confounder adjustment. Little evidence was found with preconception retinal arteriolar or venular caliber, nor venular fractal dimension and venular branching angle.

**Table 2:** Associations between retinal vascular parameters and maternal lipids using linear mixed models

Outcome variables at different time points	Retinal arteriolar caliber per SD increase (8.0 μm)	Retinal venular caliber per SD increase (12.2 μm)	Fractal dimension arteriole per SD increase (0.04 df)	Fractal dimension venule per SD increase (0.04 df)	Branching angle arteriole per SD increase (7.9°)	Branching angle venule per SD increase (8.0 °)
Total cholesterol						
Model 1	0.05(−0.09, 0.18)	0.06(−0.08, 0.2)	−0.04(−0.18, 0.1)	−0.02(−0.16, 0.11)	**0.14(0, 0.27)**	0.02(−0.12, 0.16)
Model 2	0.07(−0.06, 0.21)	0.11(−0.03, 0.25)	−0.02(−0.16, 0.12)	0(−0.14, 0.13)	**0.15(0.01, 0.28)**	0(−0.14, 0.13)
Triglycerides						
Model 1	0(−0.12, 0.12)	0.08(−0.03, 0.2)	**0.12(0.01, 0.24)**	−0.01(−0.13, 0.11)	−0.01(−0.13, 0.11)	−0.09(−0.2, 0.03)
Model 2	−0.06(−0.17, 0.06)	0.01(−0.11, 0.13)	0.06(−0.05, 0.18)	−0.05(−0.16, 0.07)	0.01(−0.1, 0.13)	−0.09(−0.21, 0.02)
High-density lipoprotein cholesterol						
Model 1	0.03(−0.1, 0.15)	−0.09(−0.22, 0.04)	−**0.20(−0.32, −0.08)**	−0.09(−0.21, 0.04)	0.02(−0.11, 0.14)	0.05(−0.08, 0.18)
Model 2	0.1(−0.01, 0.21)	0.01(−0.11, 0.13)	−**0.14(−0.25, −0.02)**	−0.05(−0.17, 0.06)	0.02(−0.1, 0.13)	0.02(−0.09, 0.14)
Low-density lipoprotein cholesterol						
Model 1	0.05(−0.09, 0.18)	0.08(−0.05, 0.22)	0(−0.13, 0.13)	0.02(−0.12, 0.15)	**0.15(0.02, 0.28)**	0.01(−0.13, 0.14)
Model 2	0.06(−0.07, 0.2)	0.12(−0.02, 0.26)	0.01(−0.13, 0.15)	0.04(−0.1, 0.17)	**0.15(0.02, 0.29)**	−0.01(−0.15, 0.13)

The bold values mean p-values <0.05. Model 1, crude model

Model 2, adjusted for age, ethnicity, education, parity, family history of diabetes, pre-pregnancy smoking status and pre-pregnancy body mass index.

### Association of preconception retinal microvasculature and categories of maternal clinical dyslipidemia from mid-pregnancy to three months postpartum

**Fig. 3:**
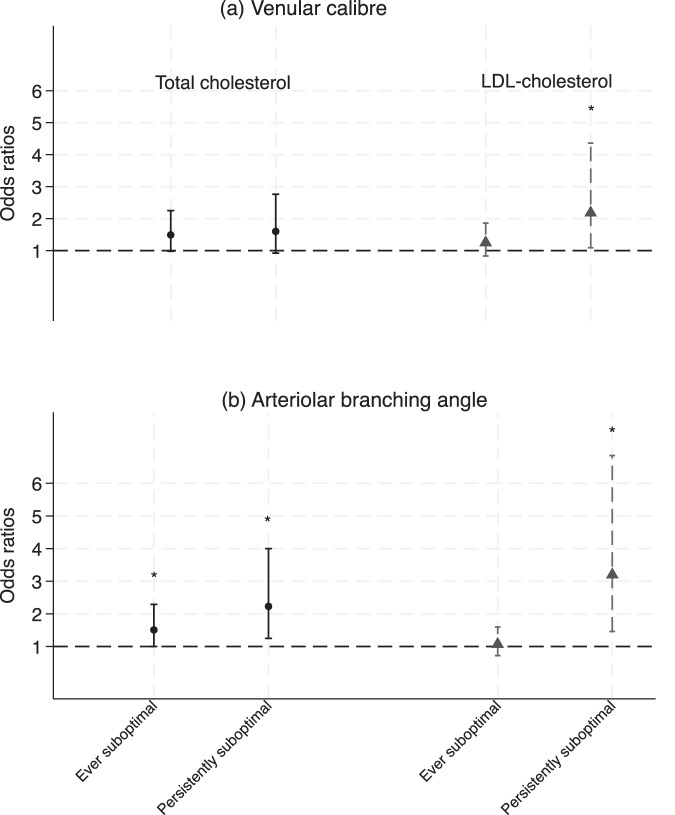
Associations between retinal vascular parameters and change of lipid level from 24 to 28 weeks of gestation to three months postpartum (Ref: normal). **a** Venular caliber, **b** Arteriolar branching angle. The circle, triangle, and hollow diamond are odds ratios for total cholesterol and low-density lipoprotein cholesterol (per SD change). Vertical bars indicate 95% confidence intervals of estimates. *indicates *p* < 0.05. The regression models were adjusted for age, ethnicity, education, family history of diabetes, pre-pregnancy smoking status, and body mass index.

**Fig. 4:**
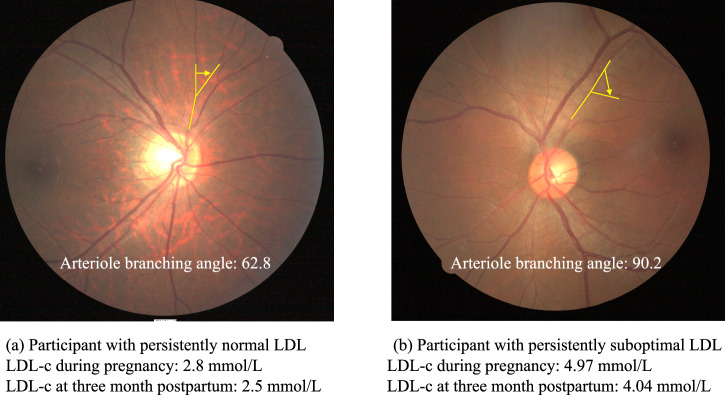
Examples of branching angle values in women with and without persistently suboptimal LDL from mid-pregnancy to 3 months postpartum. **a** shows a woman with optimal LDL. **b** shows a woman with persistently suboptimal LDL. Only one representative branch of each image is shown. The values of the branching angle represent the average value of the measurement area.

We noted that preconception retinal venular caliber (per SD increase) was associated with higher risks of persistently suboptimal LDL-C (OR = 2.18, 95% CI 1.09, 4.36), but not ever suboptimal total cholesterol (OR = 1.25, 95% CI 0.83, 1.86) after adjusting for confounding variables, see Fig. 3 and Supplementary Table 4. Arteriole branching angle (per SD increase) was associated with over two times higher risks of persistently suboptimal total cholesterol (OR = 2.23, 95% CI 1.25, 4.00) and persistently suboptimal LDL-C (OR = 3.20, 95% CI 1.46, 6.85). It should be noted that, due to the limited sample size and outcome events, the confidence intervals were wide. We provided a retinal image in Fig. 4 showing women with optimal LDL-C and persistently suboptimal LDL-C.

### Sensitivity analysis

When repeating all analyses with further adjustment for fasting glucose, high-sensitivity CRP, corresponding lipids measured before pregnancy and obstetric history of GDM, only corresponding lipid measures before pregnancy significantly attenuated the associations for fractal dimension, retinal arteriolar caliber and branching angle venule, but not venular caliber or branching angle arterioles (Supplementary Table 5–7). There were no significant interactions with maternal pre-pregnancy BMI, maternal age and ethnicity (Supplementary Notes 1–3).

## Discussion

In this study, participants were followed from preconception to three months postpartum, and we found that wider retinal venular caliber was associated with higher LDL-C at three months postpartum, but not during pregnancy, while arteriole geometrical measures, i.e., fractal dimension and branching angle, can reflect the variations of HDL-C, total cholesterol and LDL-C from pregnancy to postpartum. Particularly, a higher arteriole branching angle was associated with higher risks of hypercholesterolemia from 24 to 28 weeks of gestation to three months postpartum.

Our study found that from mid-pregnancy to three months postpartum, only half of the women had optimal lipid profiles, while the rest of the women had a suboptimal lipid profile requiring further attention. It has been shown that maternal dyslipidemia during pregnancy may signal future dyslipidemia risk in the short and long term. For example, Dunietz et al.^14^ demonstrated that high maternal lipids (LDL-C, total cholesterol, and triglycerides) and low levels of HDL-C in mid-pregnancy are associated with increased risk of dyslipidemia 7–15 years after pregnancy, independent of demographics and other health characteristics. In severe conditions, i.e., MSPHC, it is associated with feto-placental endothelial dysfunction^15,16^, pro-atherogenic lipid alterations in the maternal circulation^17^. Moreover, MSPHC was shown to be associated with early development of atherosclerosis in childhood^18^, and higher severity of myocardial infarction in adulthood of the offspring^19^. Given the adverse impacts of maternal dyslipidemia on both short- and long- term health of mothers and their offspring, early risk stratification at the preconception period represents a critical window. Early identification via non-invasive retinal imaging allows clinicians to prioritize them for formal lipid screening and lifestyle interventions before pregnancy-induced metabolic changes occur. Since persistent prenatal dyslipidemia is linked to long-term CVD risk, early detection also facilitates the transition to postpartum cardiovascular health management.

The retinal imaging and microvascular parameters provide a way forward. It has also been reported that adverse retinal arteriolar geometric morphology mirrored suboptimal cardiac structural alteration: subclinical cardiac structure was associated with a larger branching angle^20^. Similarly, our study observed that a greater arteriole branching angle was associated with higher total cholesterol and LDL-C. It is consistent with the idea that a larger retinal branching angle reflects greater workload and energy spent in maintaining efficient blood circulation^21^. Based on our findings, larger retinal branching angles may reflect higher cholesterol levels. The underlying pathophysiology between retinal geometric morphology and risks of dyslipidemia could be due to endothelial dysfunction in the suboptimal metabolic health of mothers^22^. Elevated levels of total cholesterol and LDL-C, particularly when supraphysiological, can lead to the accumulation of oxidized LDL within the vascular wall^22^. This process triggers the production of reactive oxygen species (ROS), which directly reduces the bioavailability of nitric oxide (NO)—a key vasodilator and protector of vascular integrity^23^. The resulting chronic endothelial dysfunction impairs the regulatory mechanisms that govern microvascular tone and structural remodeling^24,25^. In the retinal microvasculature, these adaptive but suboptimal changes are reflected in geometric alterations^24,25^. For instance, a larger branching angle is thought to represent a “less-than-optimal” vascular network that requires higher energy expenditure and increased shear stress to maintain blood flow^20^.

Based on the literature, fractal dimension captures morphological complexity and could serve as a dynamic marker of microvasculature^26^. Current findings supported that retinal fractal dimension may exhibit a U-shaped association with cardiovascular health, with both lower and higher values being unfavorable^27,28^. In a cross-sectional study of 407 adults (mean age 57.1 years), Joong et al.^27^ found that higher cardio-ankle vascular index, an index of arterial stiffness, was associated with decreased arteriole fractal dimension (adjusted OR 4.21 × 10^−4^, 95% CI, 2.32 × 10^−7^, 0.77). Similar evidence also showed that a reduction in the arteriole fractal dimension was associated with a higher risk of stroke^26^. In contrast, in 392 adults (mean age 67.2 years), Cheung et al.^28^ reported that a higher retinal fractal dimension was independently associated with 85% increased risk of lacunar stroke (95% CI 1.20, 2.84). In our multi-ethnic Asian population with low-risk pregnancies, we observed that a greater arteriole fractal dimension was associated with lower HDL-C during mid-to-late pregnancy and after delivery. As low HDL-C is known to impair endothelial function and promote oxidative stress, the greater fractal dimension in this context may reflect early maladaptive microvascular adaptation, aligned with what was suggested by Cheung et al.^23,28,29^.

Huang et al.^20^ reported that adverse cardiac structure was associated with arteriole geometric parameters, not venular geometry; also, Yusufu et al.^30^ reported that 18 retinal vascular measurements were significantly linked to all-cause mortality, with 16 originating from arteries. Similar to previous evidence, we did not observe associations between retinal venular geometric parameters and lipid concentrations. This could indicate that arterioles are more sensitive to variations in lipid concentrations than venules.

Adjustment for preconception lipid concentrations attenuated several retinal–lipid associations, indicating that retinal microvascular features partly reflect baseline metabolic status. However, venular caliber and arteriolar branching angle remained associated with antenatal and postpartum maternal lipid levels after adjustment, suggesting that certain retinal geometric parameters may capture microvascular information beyond that conveyed by circulating lipids alone. Nevertheless, these findings should be interpreted cautiously: rather than demonstrating strong independent predictive capacity, retinal microvascular geometry may represent an integrated vascular phenotype reflecting both metabolic and endothelial status. Formal evaluation of incremental predictive value beyond conventional risk factors is still required before any clinical application can be considered.

The American Heart Association in 2021 incorporated pregnancy complications, including GDM and hypertensive disorders, into CVD risk profiles^31^. Maternal dyslipidemia, however, remains understudied, and lipid testing is not routine in most perinatal care globally^7^. Our research team has proven the use of retinal imaging in monitoring female reproductive health^11^. Retinal imaging is a noninvasive, quick, and relatively low-cost measure that can be performed without blood sampling. This makes it particularly valuable in low-resource or rural settings where laboratory testing may be limited due to cost, infrastructure, accessibility barriers or even religious concern. A handheld retinal camera could serve a complementary role instead of multiple invasive blood draws, or it could be integrated into existing preconception or antenatal eye examinations.

Our results showed that a one-SD increase in arteriolar branching angle (7.9°) corresponded to an approximate 0.15 SD increase in LDL-C. Given the SD of LDL-C in our cohort (~0.8 mmol/L), this translates to an absolute difference of approximately 1.2 mmol/L. In a large cohort study (*n* = 730,236; follow-up 6.7 years), a 1 mmol/L increase in LDL-C was associated with a 25% higher risk of coronary heart disease (HR = 1.25, 95% CI 1.24–1.29)^32^. Although retinal microvascular parameters are not diagnostic tools and do not replace conventional lipid testing, they may offer adjunctive value for risk classification. These non-invasive “red flag” markers can be seen during routine eye exams and may prompt lipid screening in people who might otherwise go unnoticed. Further research in Asian populations is essential to validate these findings and determine whether tailored interventions are warranted.

Specifically, it could be integrated into existing preconception or antenatal eye examinations—which may already be performed for other indications—to offer a noninvasive “opportunistic” screening tool. Also, with technology and artificial intelligence putting retinal imaging into clinical and population research, microvascular status could potentially enter precision prevention for cardiometabolic diseases^33^.

Using a prospective cohort design, our study recruited women who were trying to conceive naturally and collected their health-related measurements before conceiving and followed them until pregnancies, which were clinically confirmed by ultrasound. Further, our study has strengths of using standardized protocols, validated assessment of retinal microvascular parameters, and comprehensive measurement of potential confounders in the preconception phase, such as pre-pregnancy BMI, high-sensitivity CRP, and fasting glucose. We measured women’s lipid concentrations before conception, during pregnancy, and postpartum, providing us with a full picture of women’s lipid trajectories over the process of preconception to postpartum. This also allows us to consider the impact of lipids from the preconception stage through to the postpartum period.

There are also limitations. The overall sample sizes were modest, especially since the number of participants with persistently suboptimal lipid profiles was relatively small, which may result in limited statistical power, unstable estimates, and potential model overfitting in logistic regression analyses. Therefore, these findings should be interpreted with caution and considered exploratory. Replication in larger cohorts with sufficient event numbers is required to confirm these associations. Our study only included women of Chinese, Indian, and Malay ethnicities. Hence, replication in larger sample sizes and more diverse cohorts is warranted to evaluate the generalizability of the findings. The retinal microvascular parameters were only measured before conception, which limited us to understand how it changes throughout conception. Potential selection bias might exist because women in this cohort had a lower conception rate (475/994 = 47.8%) than in other studies. It could be because women who have lower fertility than the general population would have a higher chance of participating^34^. Approximately half of the eligible participants were included in the final analysis due to incomplete follow-up. Although baseline characteristics were largely similar between included and excluded participants, the possibility of selection bias due to attrition cannot be excluded. Given the observational nature of this secondary analysis, residual or unmeasured confounding may be present. For instance, data on mothers’ medications or nutritional supplement intake, or on a personal history of dyslipidemia that could influence lipid variation, were not available. In the absence of validated postpartum-specific lipid thresholds, we applied standard non-pregnant clinical cut-offs at three months postpartum. This may have misclassified some women as having suboptimal lipids due to transient postpartum physiology, likely biasing associations toward the null^35^. Thus, the observed links between retinal features and persistent suboptimal lipid profiles likely represent conservative estimates. Nevertheless, the dynamic nature of postpartum metabolism should be considered when interpreting persistent dyslipidemia classification.

In this study, we found that, except for retinal arteriolar caliber, other retinal vascular parameters showed significant associations with maternal lipid profile during pregnancy and at three months postpartum. Using retinal imaging techniques can inform women with a high risk of suboptimal lipid profiles during pregnancy and even postpartum.

## Methods

### Study design and participants

Data were collected from the Singapore PREconception Study of long-Term maternal and child Outcomes (SPRESTO, ClinicalTrials.gov, NCT03531658). Between February 2015 and October 2017, non-pregnant Chinese, Malay, and Indian women or any combination of these three ethnicities, aged 18–45 years, who planned to conceive within 1 year from recruitment and reside in Singapore for the next five years were enrolled from Singapore KK Women’s and Children’s Hospital. The exclusion criteria were (1) already pregnant or breastfeeding at the first screening visit, (2) taking oral steroids or has type 1 or 2 diabetes, (3) taken medicine for Human Immunodeficiency Virus (HIV), Hepatitis B or C in the month before enrollment, (4) had already been trying to conceive for over 18 months and (5) have sought assisted fertility treatment (except clomiphene and letrozole) or undergone hormonal contraception treatment in the past month prior to enrollment. Details of this study have been published^36^.

The study was conducted according to the guidelines under the Declaration of Helsinki and approved by the SingHealth Centralized Institute Review Board (2014/629/D). All participants provided written informed consent.

### Exposures: preconception retinal microvascular parameters

During the preconception screening, trained photographers performed retinal examination using a non-mydriatic retinal camera (45-degree, Canon CR-1, 40D SLR digital retinal camera backing, Canon Inc.). Retinal photographs were obtained by a trained research assistant using a standardized imaging protocol. The photographer underwent formal training and certification in non-mydriatic fundus photography at the Singapore Eye Research Institute (SERI) prior to data collection. Two retinal photographs centered on the optic disc and macula of each eye were taken without pharmacological pupillary dilation. All images were graded at the SERI Ocular Epidemiology Research Grading Centre, where the semi-automated computer-based program (Singapore I Vessel Assessment [SIVA] version 4.0, Singapore Eye Research Institute) was developed. Image analysis was conducted by professional graders who were masked to participants’ clinical characteristics and lipid measurements. Standardized grading protocols were applied throughout the study period. The graders assessed all retinal vessels beyond 25 μm in width crossing through 0.5–2.0 disc diameters from the optic disc margin. Within the 628 participating women, 605 retinal images were successfully graded. The gradability rate is 96.3%.

The retinal arteriolar and venular caliber (μm) are defined as the average width of retinal arterioles and venules, respectively. Fractal dimension without a specific unit quantifies the complexity of the branching pattern of the retinal vascular tree, which was calculated from the outlined retinal vessels using the box-counting method. The branching angle with a unit of degree is defined as the first angle subtended between two daughter vessels at each bifurcation. Inter-grader and intra-grader reliability were assessed in 10% of randomly selected retinal photographs, and the interclass and intraclass correlation coefficients ranged between 0.94 and 0.99 for retinal arteriolar and venular caliber, between 0.85 and 0.94 for vascular fractal dimension and branching angle, as previously reported and validated^37^. Oversight of retinal image acquisition, grading procedures, and quality assurance was conducted in collaboration with investigators experienced in retinal vascular epidemiology.

### Outcomes: maternal lipid assessments from mid-pregnancy to 3 months postpartum

Fasting blood samples were collected at recruitment, 24–28 weeks of gestation and at three months postpartum visit, respectively. Maternal venous blood was collected into silicone-coated tubes, and serum was obtained by centrifugation at 1600 × *g* for 10 min at 4 °C. The serum was stored at −80 °C until sample batch analysis at the National University Hospital clinical laboratory. Using a Beckman AU5800 analyzer, TG was measured by colorimetric assays, while total cholesterol, LDL-C, and HDL-C were measured by colorimetric and enzymatic methods, respectively. According to the clinical practice guidelines of Singapore^38^, we defined total cholesterol ≥5.2 mmol/L, triglycerides ≥2.3, HDL-C < 1.0 mmol/L, LDL-C ≥ 3.4 mmol/L as suboptimal lipid levels, but during pregnancy, the suboptimal total cholesterol and LDL-C were defined as ≥7.5 mmol/L and ≥4.4 mmol/L, respectively. At three months postpartum, non-pregnant lipid thresholds were applied in accordance with national clinical guidelines, as postpartum-specific cut-offs are not currently established. We defined optimal lipid profile as women with normal lipids from 24 to 28 weeks of gestation to three months postpartum, ever suboptimal lipids as women with suboptimal lipids at either time point, and persistently suboptimal lipids if lipid profiles were suboptimal at both time points.

### Covariates

During recruitment, trained research coordinators used questionnaires to collect information about sociodemographic factors and health history. The following data were collected: maternal age, ethnicity (Chinese, Malay, Indian, and others), college education (yes versus no), parity (nulliparous versus parous), preconception smoking status (yes versus no), family history of diabetes (yes versus no), and obstetric history of GDM (yes versus no). They also measured participants’ height and weight. Weight was measured to the nearest 0.1 kg (SECA 803) and height to the nearest 0.1 cm (SECA 213). All measurements were taken in duplicate, and the average was used. Body mass index (BMI) was derived using weight divided by height squared (kg/m^2^).

At the recruitment (preconception) visit, overnight fasting blood was collected from women, and plasma fasting glucose was measured using the ARCHITECT c8000 Clinical Chemistry Analyzer (Abbott Laboratories). All samples were kept at 4 °C, immediately sent to the hospital laboratory, centrifuged within 30 min, and analyzed within 1 hour from the earliest blood draw.

### Statistical analyses

The normality of data was checked via sktest. We performed analyses in the following steps: (1) we fit linear regression models to examine associations between preconception retinal microvascular parameters and maternal lipids measured at 24–28 weeks of gestation and at three months postpartum; (2) using linear mixed models, we examined if preconception retinal microvascular parameters were associated with maternal lipids across pregnancy and three months postpartum; (3) using logistic regression model, we examined if preconception retinal microvascular parameters were associated with categories of maternal clinical dyslipidemia from mid-pregnancy to three months postpartum (i.e., optimal, ever suboptimal and persistently suboptimal lipid profile from pregnancy to postpartum).

In all regressions, we applied both unadjusted and adjusted models. Model 1 was an unadjusted model; Model 2 estimates were adjusted for maternal age, ethnicity, education levels, parity, family history of diabetes, pre-pregnancy smoking status and pre-pregnancy BMI. In sensitivity analysis, models were further adjusted for each of the following covariates (i) fasting glucose, high-sensitivity CRP, corresponding lipid concentrations measured at preconception and obstetric history of GDM, given only half of the participating women provided this information; (ii) interactions between exposures with maternal age, ethnicity and pre-pregnancy BMI were investigated. The potential confounders were defined a *priori*. For all analyses, we internally standardized exposures and/or outcomes (i.e., a mean of zero and a standard deviation [SD] of 1) to present standardized regression coefficients. All statistical analyses were performed in Stata 17.0 SE (StataCorp LP, TX, USA), and the results were presented with either standardized β or odds ratio (OR) with 95% confidence intervals (CIs). Two-tailed *P* values were reported.

## Supplementary information

Supplementary information

## Data Availability

The data described in the manuscript and code book will be available upon request, pending the application and approval of a data-sharing agreement.

## Code availability

The analytic code will be available upon request, pending the application and approval of a code-sharing agreement.
