# Supplementary material for: Associations of preconception retinal microvasculature with lipid profiles from mid-pregnancy to three months postpartum
Source: NPJ Cardiovasc Health. 2026 May 15;3:29. doi: 10.1038/s44325-026-00130-9 (PMC13230875; doi:10.1038/s44325-026-00130-9)
Supplement: Supplementary file 1 — Supplementary information [file 44325_2026_130_MOESM1_ESM.docx]

**Supplementary Table 1. Sample characteristics**

| **Variables** | **Mean (SD)/ N(%)** |
| --- | --- |
| Mother age (whole years) | 29.9 (3.2) |
| Ethnicity |  |
| Chinese | 74.7 |
| Malay | 15.2 |
| Indian | 8.9 |
| Others | 1.2 |
| Highest education |  |
| Below university | 29.8 |
| University and above | 70.2 |
| Nulliparous (yes, %) | 60.8 |
| Pre-pregnancy smoking (yes, %) | 8.9 |
| Pre-pregnancy BMI (kg/m^2^) | 22.82 (4.06) |
| Gestational weeks (week) | 39.2 (2.8) |
| Total gestational weight gain (kg) | 11.2 (4.3) |
| Fasting glucose (mmol/L) | 4.7 (0.4) |
| Lipid profiles—before conception (mmol/L) |  |
| Total cholesterol | 4.7 (0.8) |
| Triglycerides | 0.8 (0.4) |
| High density lipoprotein cholesterol | 1.4 (0.3) |
| Low density lipoprotein cholesterol | 2.9 (0.7) |
| Lipid profiles—at 26-28 weeks of gestation (mmol/L) |  |
| Total cholesterol | 6.4 (1.0) |
| Triglycerides | 2.0 (0.6) |
| High density lipoprotein cholesterol | 2.0 (0.4) |
| Low density lipoprotein cholesterol | 3.5 (0.8) |
| Lipid profiles—three months postpartum (mmol/L) |  |
| Total cholesterol | 5.1 (0.9) |
| Triglycerides | 0.8 (0.4) |
| High density lipoprotein cholesterol | 1.6 (0.3) |
| Low density lipoprotein cholesterol | 3.1 (0.8) |
| Preconception Retinal arteriolar calibre (μm) | 119.1 (8.3) |
| Preconception Retinal venular calibre (μm) | 171.6 (12.5) |
| Preconception Fractal dimension arteriole | 1.6 (0.04) |
| Preconception Fractal dimension venule | 1.2 (0.04) |
| Preconception Branching angle arteriole | 79.5 (7.7) |
| Preconception Branching angle venule | 79.3 (7.9) |

sTable 2. Compare baseline characteristics of included and excluded participant

| **Variables** | **Included**  **(n=187)** | **Excluded**  **(n=179)** | **p-value** |
| --- | --- | --- | --- |
|  | **Mean (SD)/ %** | **Mean (SD)/ %** |  |
| Mother age (whole years) | 29.9 (3.2) | 29.4 (3.1) | 0.56 |
| Ethnicity |  |  |  |
| Chinese | 74.7 | 76.4 | 0.05 |
| Malay | 15.2 | 13.3 |  |
| Indian | 8.9 | 4.2 |  |
| Others | 1.2 | 6.1 |  |
| Highest education |  |  | 0.20 |
| Below university | 29.8 | 23.5 |  |
| University | 70.2 | 76.5 |  |
| Nulliparous (yes, %) | 60.8 | 64.5 | 0.49 |
| Pre-pregnancy smoking (yes, %) | 8.9 | 6.0 | 0.61 |
| Pre-pregnancy BMI (kg/m^2^) | 22.82 (4.06) | 23.01 (4.23) | 0.68 |
| Lipid profiles—before conception (mmol/L) |  |  |  |
| Total cholesterol | 4.68 (0.81) | 4.73 (0.81) | 0.55 |
| Triglycerides | 0.8 (0.37) | 0.79 (0.36) | 0.76 |
| High density lipoprotein cholesterol | 1.43 (0.28) | 1.5 (0.28) | 0.04 |
| Low density lipoprotein cholesterol | 2.86 (0.69) | 2.87 (0.72) | 0.86 |
| Preconception Retinal arteriolar calibre (μm) | 119.13 (8.34) | 118.97 (6.18) | 0.92 |
| Preconception Retinal venular calibre (μm) | 171.61 (12.53) | 169.65 (9.82) | 0.43 |
| Preconception Fractal dimension arteriole | 1.25 (0.04) | 1.24 (0.04) | 0.59 |
| Preconception Fractal dimension venule | 1.22 (0.04) | 1.22 (0.03) | 0.94 |
| Preconception Branching angle arteriole | 79.52 (7.67) | 78.37 (9.19) | 0.47 |
| Preconception Branching angle venule | 79.33 (7.92) | 80.61 (7.48) | 0.42 |

**Supplementary Table 3. Associations between preconception retinal vascular parameters and maternal lipids measured during pregnancy and three-month postpartum (Standardised beta and 95%CI are reported)**

| **Outcome variables** | **Retinal arteriolar calibre**  **Per SD increase (8.0 um)** | **Retinal venular calibre**  **Per SD increase (12.2 um)** | **Fractal dimension arteriole**  **Per SD increase (0.04 df)** | **Fractal dimension venule**  **Per SD increase (0.04 df)** | **Branching angle arteriole**  **Per SD increase (7.9 degree)** | **Branching angle venule**  **Per SD increase (8.0 degree)** |
| --- | --- | --- | --- | --- | --- | --- |
| **During pregnancy (24-28 weeks of gestation)** | | | | | | |
| Total cholesterol | |  |  |  |  |  |
| Model 1 | 0.00 (-0.15, 0.16) | 0.01 (-0.14, 0.16) | -0.06 (-0.21, 0.09) | -0.04 (-0.19, 0.11) | **0.15 (0.00, 0.31)** | 0.04 (-0.11, 0.19) |
| Model 2 | 0.04 (-0.12, 0.20) | 0.06 (-0.10, 0.22) | -0.04 (-0.20, 0.12) | -0.03 (-0.19, 0.13) | **0.17 (0.01, 0.32)** | 0.04 (-0.11, 0.20) |
| Triglycerides |  |  |  |  |  |  |
| Model 1 | -0.02 (-0.16, 0.12) | 0.04 (-0.10, 0.18) | 0.12 (-0.02, 0.27) | -0.02 (-0.17, 0.12) | 0.02 (-0.13, 0.16) | -0.09 (-0.23, 0.05) |
| Model 2 | -0.08 (-0.22, 0.07) | -0.02 (-0.17, 0.13) | 0.10 (-0.05, 0.25) | -0.02 (-0.17, 0.13) | 0.03 (-0.11, 0.18) | -0.10 (-0.24, 0.05) |
| High-density lipoprotein cholesterol  Model 1 | -0.03 (-0.18, 0.12) | -0.11 (-0.26, 0.04) | **-0.20 (-0.35, -0.05)** | -0.06 (-0.21, 0.09) | -0.03 (-0.18, 0.12) | 0.11 (-0.04, 0.26) |
| Model 2 | 0.04 (-0.10, 0.19) | -0.02 (-0.17, 0.13) | **-0.16 (-0.31, -0.02)** | -0.05 (-0.19, 0.10) | -0.02 (-0.16, 0.12) | 0.10 (-0.04, 0.24) |
| Low-density lipoprotein cholesterol  Model 1 | 0.03 (-0.12, 0.18) | 0.04 (-0.11, 0.19) | -0.03 (-0.18, 0.12) | -0.01 (-0.16, 0.14) | 0.19 (0.04, 0.34) | 0.02 (-0.13, 0.17) |
| Model 2 | 0.06 (-0.10, 0.21) | 0.08 (-0.08, 0.24) | -0.02 (-0.17, 0.14) | -0.00 (-0.16, 0.16) | **0.19 (0.04, 0.35)** | 0.02 (-0.14, 0.17) |
| **Three-month after birth** |  |  |  |  |  |  |
| Total cholesterol |  |  |  |  |  |  |
| Model 1 | 0.10 (-0.06, 0.26) | **0.18 (0.02, 0.34)** | 0.01 (-0.15, 0.18) | 0.05 (-0.12, 0.21) | 0.20 (0.03, 0.37) | -0.04 (-0.20, 0.13) |
| Model 2 | 0.10 (-0.06, 0.26) | **0.21 (0.04, 0.37)** | 0.03 (-0.14, 0.20) | 0.10 (-0.06, 0.27) | **0.20 (0.04, 0.37)** | -0.09 (-0.26, 0.08) |
| Triglycerides |  |  |  |  |  |  |
| Model 1 | 0.02 (-0.12, 0.17) | 0.16 (0.02, 0.31) | 0.12 (-0.03, 0.27) | 0.03 (-0.12, 0.17) | -0.03 (-0.19, 0.12) | -0.10 (-0.24, 0.05) |
| Model 2 | -0.03 (-0.17, 0.10) | 0.08 (-0.06, 0.22) | 0.03 (-0.12, 0.17) | -0.02 (-0.16, 0.11) | -0.01 (-0.15, 0.13) | -0.09 (-0.24, 0.05) |
| High density lipoprotein cholesterol |  |  |  |  |  |  |
| Model 1 | 0.11 (-0.04, 0.25) | -0.04 (-0.19, 0.11) | -0.19 (-0.33, -0.04) | -0.11 (-0.26, 0.04) | 0.11 (-0.04, 0.27) | -0.02 (-0.17, 0.13) |
| Model 2 | **0.17 (0.04, 0.30)** | 0.04 (-0.10, 0.18) | -0.10 (-0.24, 0.04) | -0.05 (-0.19, 0.09) | 0.09 (-0.05, 0.23) | -0.08 (-0.22, 0.06) |
| Low density lipoprotein cholesterol |  |  |  |  |  |  |
| Model 1 | 0.06 (-0.09, 0.22) | 0.18 (0.03, 0.34) | 0.07 (-0.10, 0.23) | 0.09 (-0.07, 0.25) | **0.18 (0.01, 0.35)** | -0.01 (-0.18, 0.15) |
| Model 2 | 0.05 (-0.12, 0.21) | **0.19 (0.03, 0.36)** | 0.07 (-0.10, 0.24) | 0.14 (-0.03, 0.31) | **0.19 (0.02, 0.36)** | -0.05 (-0.22, 0.12) |

Model 1, crude model

Model 2, adjusted for age, ethnicity, education, parity, family history of diabetes, pre-pregnancy smoking status and body mass index.

**Supplementary Table 4. Associations between retinal vascular parameters and categories of maternal clinical dyslipidemia from 24-28 weeks of gestation to three-month postpartum**

| **Suboptimal outcomes** | **Retinal arteriolar calibre**  **Per SD increase (8.0 um)** | **Retinal venular calibre**  **Per SD increase (12.2 um)** | | **Fractal dimension arteriole**  **Per SD increase (0.04 df)** | | | **Fractal dimension venule**  **Per SD increase (0.04 df)** | | **Branching angle arteriole**  **Per SD increase (7.9 degree)** | | **Branching angle venule**  **Per SD increase (8.0 degree)** |  |
| --- | --- | --- | --- | --- | --- | --- | --- | --- | --- | --- | --- | --- |
| **Total cholesterol** | | | | | | | | | | | |  |
| **Model 1** |  |  | |  | |  | |  | |  | |  |
| Normal | Ref | Ref | | Ref | | Ref | | Ref | | Ref | |  |
| Ever suboptimal | 1.27(0.90, 1.81) | 1.37(0.95, 1.97) | | 0.80(0.56, 1.14) | | 0.87(0.61, 1.23) | | 1.42(0.96, 2.09) | | 1.18(0.82, 1.71) | |  |
| Persistently suboptimal | 1.20(0.74, 1.95) | 1.46(0.88, 2.43) | | 0.92(0.56, 1.51) | | 0.98(0.60, 1.60) | | 2.20(1.25, 3.87) | | 1.12(0.67, 1.86) | |  |
| **Model 2** |  |  | |  | |  | |  | |  | |  |
| Ever suboptimal | 1.28(0.87, 1.89) | 1.49(0.98, 2.25) | | 0.78(0.53, 1.15) | | 0.97(0.66, 1.43) | | **1.51(1.00, 2.29)** | | 1.16(0.77, 1.74) | |  |
| Persistently suboptimal | 1.26(0.76, 2.10) | 1.60(0.92, 2.76) | | 0.88(0.52, 1.50) | | 1.00(0.58, 1.72) | | **2.23(1.25, 4.00)** | | 1.15(0.66, 1.98) | |  |
| **LDL cholesterol** |  |  | |  | |  | |  | |  | |  |
| **Model 1** |  |  | |  | |  | |  | |  | |  |
| Normal | Ref | | Ref | | Ref | | Ref | | Ref | | Ref | |
| Ever suboptimal | 1.27(0.90, 1.81) | | 1.34(0.93, 1.93) | | 1.02(0.72, 1.46) | | 1.16(0.81, 1.66) | | 1.02(0.70, 1.50) | | 1.24 (0.86, 1.80) | |
| Persistently suboptimal | 1.24(0.70, 2.20) | | 1.85(0.99, 3.44) | | 0.94(0.52, 1.69) | | 1.15(0.64, 2.08) | | 3.19(1.54, 6.63) | | 0.91(0.50, 1.66) | |
| **Model 2** |  | |  | |  | |  | |  | |  | |
| Ever suboptimal | 1.20(0.83, 1.75) | | 1.25(0.83, 1.86) | | 0.93(0.64, 1.36) | | 1.22(0.83, 1.80) | | 1.07(0.72, 1.60) | | 1.28(0.85, 1.92) | |
| Persistently suboptimal | 1.38(0.73, 2.63) | | **2.18(1.09, 4.36)** | | 0.94(0.50, 1.79) | | 1.23(0.66, 2.28) | | **3.20(1.46, 6.85)** | | 0.93(0.50, 1.73) | |

Model 1, crude model

Model 2, adjusted for age, ethnicity, education, parity, family history of diabetes, pre-pregnancy smoking status and body mass index

**Supplementary Table 5. Sensitivity analysis of associations between preconception retinal vascular parameters and maternal lipids measured during pregnancy and three-month postpartum**

| **Outcome variables** | **Retinal arteriolar calibre**  **Per SD increase (8.0 um)** | **Retinal venular calibre**  **Per SD increase (12.2 um)** | **Fractal dimension arteriole**  **Per SD increase (0.04 df)** | **Fractal dimension venule**  **Per SD increase (0.04 df)** | **Branching angle arteriole**  **Per SD increase (7.9 degree)** | **Branching angle venule**  **Per SD increase (8.0 degree)** |
| --- | --- | --- | --- | --- | --- | --- |
| **Sensitivity analysis 1: Model 2+ fasting glucose** | | | | | | |
| **During pregnancy (24-28 weeks of gestation, n = 178)** | |  |  |  |  |  |
| Total cholesterol | 0.04 (-0.11, 0.19) | 0.06 (-0.10, 0.23) | -0.04 (-0.20, 0.12) | -0.04 (-0.20, 0.12) | **0.17 (0.02, 0.32)** | 0.04 (-0.12, 0.21) |
| Triglycerides | -0.08 (-0.23, 0.07) | -0.04 (-0.19, 0.12) | 0.12 (-0.04, 0.27) | -0.00 (-0.15, 0.15) | 0.04 (-0.11, 0.18) | -0.12 (-0.27, 0.04) |
| High-density lipoprotein cholesterol | 0.04 (-0.10, 0.19) | -0.01 (-0.16, 0.14) | **-0.16 (-0.31, -0.02)** | -0.06 (-0.20, 0.09) | -0.03 (-0.17, 0.12) | 0.10 (-0.05, 0.25) |
| Low-density lipoprotein cholesterol | 0.06 (-0.10, 0.22) | 0.09 (-0.08, 0.25) | -0.02 (-0.18, 0.15) | -0.01 (-0.18, 0.15) | **0.20 (0.05, 0.35)** | 0.02 (-0.14, 0.19) |
| **Three-month after birth (n = 151)** |  |  |  |  |  |  |
| Total cholesterol | 0.10 (-0.06, 0.26) | **0.21 (0.05, 0.38)** | 0.05 (-0.13, 0.22) | 0.11 (-0.06, 0.27) | **0.20 (0.04, 0.37)** | -0.09 (-0.26, 0.09) |
| Triglycerides | -0.04 (-0.17, 0.10) | 0.09 (-0.05, 0.23) | 0.06 (-0.08, 0.21) | -0.01 (-0.15, 0.12) | -0.02 (-0.16, 0.12) | -0.07 (-0.21, 0.08) |
| High density lipoprotein cholesterol | **0.17 (0.03, 0.30)** | 0.05 (-0.10, 0.19) | -0.10 (-0.25, 0.05) | -0.04 (-0.18, 0.10) | 0.09 (-0.05, 0.23) | -0.09 (-0.24, 0.05) |
| Low density lipoprotein cholesterol | 0.05 (-0.12, 0.21) | **0.20 (0.03, 0.37)** | 0.08 (-0.10, 0.26) | 0.14 (-0.03, 0.31) | **0.19 (0.02, 0.36)** | -0.05 (-0.22, 0.13) |
| **Sensitivity analysis 2: Model 2+ high-sensitivity C-reactive protein** | | | | | | |
| **During pregnancy (24-28 weeks of gestation, n = 175)** | |  |  |  |  |  |
| Total cholesterol | 0.04 (-0.13, 0.20) | 0.06 (-0.12, 0.24) | -0.04 (-0.21, 0.13) | -0.05 (-0.21, 0.12) | **0.17 (0.01, 0.33)** | 0.06 (-0.11, 0.22) |
| Triglycerides | -0.09 (-0.24, 0.06) | -0.06 (-0.22, 0.10) | **0.16 (0.01, 0.32)** | 0.02 (-0.14, 0.17) | 0.02 (-0.13, 0.16) | -0.12 (-0.27, 0.03) |
| High-density lipoprotein cholesterol | 0.05 (-0.10, 0.20) | -0.01 (-0.17, 0.15) | **-0.18 (-0.33, -0.03)** | -0.06 (-0.21, 0.09) | -0.03 (-0.18, 0.11) | 0.13 (-0.01, 0.28) |
| Low-density lipoprotein cholesterol | 0.05 (-0.11, 0.22) | 0.09 (-0.09, 0.26) | -0.03 (-0.20, 0.14) | -0.03 (-0.19, 0.14) | **0.20 (0.05, 0.36)** | 0.03 (-0.14, 0.20) |
| **Three-month after birth (n = 149)** |  |  |  |  |  |  |
| Total cholesterol | 0.06 (-0.11, 0.23) | 0.16 (-0.02, 0.35) | 0.07 (-0.11, 0.24) | 0.10 (-0.06, 0.27) | **0.18 (0.01, 0.35)** | -0.08 (-0.25, 0.10) |
| Triglycerides | -0.05 (-0.19, 0.09) | 0.07 (-0.08, 0.23) | 0.06 (-0.09, 0.21) | -0.02 (-0.16, 0.12) | -0.03 (-0.17, 0.12) | -0.09 (-0.24, 0.06) |
| High-density lipoprotein cholesterol | 0.12 (-0.01, 0.26) | -0.03 (-0.18, 0.13) | -0.10 (-0.25, 0.04) | -0.06 (-0.19, 0.08) | 0.08 (-0.07, 0.22) | -0.07 (-0.22, 0.07) |
| Low-density lipoprotein cholesterol | 0.03 (-0.15, 0.20) | 0.17 (-0.01, 0.36) | 0.10 (-0.08, 0.28) | 0.14 (-0.03, 0.31) | **0.18 (0.00, 0.35)** | -0.03 (-0.21, 0.14) |
| **Sensitivity analysis 3: Model 2+ corresponding lipids** | | | | | | |
| **During pregnancy (24-28 weeks of gestation, n = 179)** | |  |  |  |  |  |
| Total cholesterol | 0.01 (-0.11, 0.13) | 0.00 (-0.12, 0.13) | -0.05 (-0.18, 0.07) | -0.08 (-0.20, 0.05) | 0.12 (-0.01, 0.24) | 0.11 (-0.01, 0.23) |
| Triglycerides | -0.05 (-0.18, 0.08) | -0.00 (-0.14, 0.13) | 0.09 (-0.04, 0.23) | -0.02 (-0.16, 0.11) | 0.04 (-0.09, 0.17) | -0.05 (-0.19, 0.08) |
| High-density lipoprotein cholesterol | -0.00 (-0.12, 0.11) | -0.04 (-0.16, 0.08) | -0.08 (-0.20, 0.04) | -0.10 (-0.22, 0.02) | 0.00 (-0.11, 0.11) | **0.13 (0.02, 0.24)** |
| Low-density lipoprotein cholesterol | 0.04 (-0.09, 0.16) | 0.02 (-0.11, 0.15) | -0.06 (-0.19, 0.07) | -0.03 (-0.16, 0.10) | **0.13 (0.01, 0.25)** | 0.07 (-0.06, 0.20) |
| **Three-month after birth (n = 152)** |  |  |  |  |  |  |
| Total cholesterol | 0.02 (-0.08, 0.13) | 0.09 (-0.02, 0.20) | -0.03 (-0.15, 0.08) | 0.03 (-0.08, 0.14) | **0.15 (0.04, 0.26)** | 0.04 (-0.07, 0.15) |
| Triglycerides | -0.03 (-0.15, 0.09) | 0.09 (-0.04, 0.21) | -0.01 (-0.13, 0.12) | -0.06 (-0.18, 0.07) | 0.00 (-0.12, 0.13) | -0.03 (-0.16, 0.10) |
| High-density lipoprotein cholesterol | **0.10 (0.00, 0.20)** | 0.00 (-0.10, 0.11) | -0.01 (-0.12, 0.10) | -0.08 (-0.19, 0.02) | **0.11 (0.01, 0.21)** | -0.07 (-0.17, 0.04) |
| Low-density lipoprotein cholesterol | -0.01 (-0.11, 0.10) | 0.07 (-0.03, 0.18) | -0.03 (-0.14, 0.08) | 0.09 (-0.01, 0.20) | **0.11 (0.00, 0.22)** | 0.07 (-0.04, 0.18) |
| **Sensitivity analysis 4: Model 2+ obstetric history of GDM** | | | | | | |
| **During pregnancy (24-28 weeks of gestation, n = 178)** | |  |  |  |  |  |
| Total cholesterol | 0.04 (-0.12, 0.20) | 0.06 (-0.10, 0.22) | -0.04 (-0.21, 0.12) | -0.04 (-0.20, 0.12) | **0.17 (0.02, 0.33)** | 0.06 (-0.10, 0.22) |
| Triglycerides | -0.08 (-0.23, 0.07) | -0.03 (-0.18, 0.12) | 0.11 (-0.05, 0.26) | -0.00 (-0.16, 0.15) | 0.04 (-0.11, 0.19) | -0.12 (-0.27, 0.03) |
| High-density lipoprotein cholesterol | 0.04 (-0.10, 0.19) | -0.02 (-0.16, 0.13) | **-0.16 (-0.31, -0.02)** | -0.06 (-0.21, 0.08) | -0.02 (-0.16, 0.12) | 0.12 (-0.03, 0.27) |
| Low-density lipoprotein cholesterol | 0.06 (-0.10, 0.22) | 0.09 (-0.08, 0.25) | -0.02 (-0.18, 0.14) | -0.02 (-0.18, 0.15) | **0.20 (0.04, 0.35)** | 0.03 (-0.13, 0.20) |
| **Three-month after birth (n = 152)** |  |  |  |  |  |  |
| Total cholesterol | 0.10 (-0.06, 0.26) | 0.21 (0.05, 0.38) | 0.03 (-0.14, 0.21) | 0.11 (-0.06, 0.28) | **0.20 (0.03, 0.37)** | -0.09 (-0.26, 0.08) |
| Triglycerides | -0.04 (-0.17, 0.10) | 0.09 (-0.05, 0.23) | 0.04 (-0.11, 0.19) | -0.02 (-0.16, 0.12) | -0.02 (-0.16, 0.12) | -0.10 (-0.24, 0.04) |
| High-density lipoprotein cholesterol | **0.16 (0.03, 0.30)** | 0.04 (-0.10, 0.18) | -0.10 (-0.24, 0.05) | -0.05 (-0.19, 0.09) | 0.09 (-0.05, 0.24) | -0.08 (-0.22, 0.06) |
| Low-density lipoprotein cholesterol | 0.05 (-0.11, 0.21) | 0.20 (0.03, 0.37) | 0.07 (-0.11, 0.25) | 0.15 (-0.02, 0.31) | **0.19 (0.02, 0.36)** | -0.05 (-0.22, 0.12) |

Model 2, adjusted for age, ethnicity, education, parity, family history of diabetes, pre-pregnancy smoking status and body mass index

**Supplementary Table 6. Sensitivity analysis of associations between retinal vascular parameters and maternal lipids using linear mixed models**

| **Outcome variables at different time points** | **Retinal arteriolar calibre**  **Per SD increase (8.0 um)** | **Retinal venular calibre**  **Per SD increase (12.2 um)** | **Fractal dimension arteriole**  **Per SD increase (0.04 df)** | **Fractal dimension venule**  **Per SD increase (0.04 df)** | **Branching angle arteriole**  **Per SD increase (7.9 degree)** | **Branching angle venule**  **Per SD increase (8.0 degree)** |
| --- | --- | --- | --- | --- | --- | --- |
| **Sensitivity analysis 1: Model 2+ fasting glucose (n = 182)** | | | | | | |
| Total cholesterol | 0.07(-0.06, 0.21) | 0.11(-0.03, 0.25) | -0.01(-0.15, 0.13) | 0.00(-0.14, 0.14) | **0.15(0.02, 0.28)** | -0.00(-0.14, 0.14) |
| Triglycerides | -0.06(-0.17, 0.05) | 0.01(-0.11, 0.13) | 0.09(-0.02, 0.21) | -0.03(-0.14, 0.09) | 0.01(-0.11, 0.12) | -0.07(-0.19, 0.04) |
| High density lipoprotein cholesterol | 0.10(-0.01, 0.21) | 0.01(-0.11, 0.13) | **-0.15(-0.26, -0.03)** | -0.06(-0.17, 0.06) | 0.02(-0.09, 0.13) | 0.02(-0.10, 0.13) |
| Low density lipoprotein cholesterol | 0.06(-0.07, 0.20) | 0.12(-0.02, 0.26) | 0.02(-0.12, 0.16) | 0.04(-0.10, 0.17) | **0.16(0.03, 0.29)** | -0.01(-0.15, 0.13) |
| **Sensitivity analysis 2: Model 2+ high-sensitivity C-reactive protein (n = 180)** | | | | | | |
| Total cholesterol | 0.07(-0.08, 0.21) | 0.10(-0.05, 0.25) | -0.01(-0.16, 0.14) | -0.01(-0.16, 0.13) | **0.14(0.00, 0.28)** | 0.02(-0.13, 0.16) |
| Triglycerides | -0.07(-0.19, 0.05) | -0.02(-0.15, 0.10) | 0.12(-0.00, 0.24) | -0.01(-0.13, 0.10) | -0.01(-0.12, 0.10) | -0.10(-0.22, 0.01) |
| High density lipoprotein cholesterol | 0.09(-0.03, 0.20) | -0.01(-0.14, 0.11) | **-0.15(-0.27, -0.03)** | -0.07(-0.19, 0.04) | 0.00(-0.11, 0.12) | 0.04(-0.08, 0.16) |
| Low density lipoprotein cholesterol | 0.06(-0.04, 0.21) | 0.13(-0.03, 0.28) | 0.01(-0.14, 0.16) | 0.02(-0.13, 0.16) | 0.16(0.02, 0.29) | 0.01(-0.13, 0.16) |
| **Sensitivity analysis 3: Model 2+ corresponding lipids (n = 184)** | | | | | | |
| Total cholesterol | 0.03(-0.06, 0.12) | 0.05(-0.04, 0.14) | -0.04(-0.13, 0.05) | -0.03(-0.11, 0.06) | **0.11(0.03, 0.20)** | 0.07(-0.02, 0.16) |
| Triglycerides | -0.04(-0.13, 0.05) | 0.04(-0.06, 0.13) | 0.05(-0.05, 0.14) | -0.04(-0.13, 0.06) | 0.02(-0.07, 0.11) | -0.05(-0.14, 0.05) |
| High density lipoprotein cholesterol | 0.05(-0.03, 0.12) | -0.02(-0.10, 0.06) | -0.05(-0.13, 0.03) | **-0.09(-0.17, -0.02)** | 0.05(-0.03, 0.12) | 0.04(-0.04, 0.12) |
| Low density lipoprotein cholesterol | 0.03(-0.06, 0.11) | 0.06(-0.03, 0.15) | -0.04(-0.13, 0.05) | 0.02(-0.07, 0.11) | 0.10(0.02, 0.19) | 0.06(-0.03, 0.15) |
| **Sensitivity analysis 4: Model 2+ obstetric history of GDM (n = 184)** | | | | | | |
| Total cholesterol | 0.07(-0.06, 0.21) | 0.12(-0.02, 0.26) | -0.06(-0.21, 0.08) | -0.01(-0.14, 0.13) | **0.14(0.01, 0.28)** | -0.01(-0.15, 0.13) |
| Triglycerides | -0.06(-0.17, 0.05) | 0.01(-0.10, 0.13) | 0.03(-0.09, 0.16) | -0.04(-0.16, 0.07) | 0.01(-0.10, 0.12) | -0.09(-0.21, 0.02) |
| High density lipoprotein cholesterol | 0.10(-0.01, 0.21) | 0.00(-0.11, 0.12) | -0.15(-0.27, -0.03) | -0.06(-0.17, 0.06) | 0.02(-0.09, 0.14) | 0.03(-0.09, 0.14) |
| Low density lipoprotein cholesterol | 0.06(-0.07, 0.20) | 0.13(-0.01, 0.27) | -0.02(-0.17, 0.12) | 0.03(-0.10, 0.17) | **0.15(0.02, 0.29)** | -0.01(-0.15, 0.13) |

Model 2, adjusted for age, ethnicity, education, parity, family history of diabetes, pre-pregnancy smoking status and body mass index

**Supplementary Table 7. Sensitivity analysis of associations between retinal vascular parameters and categories of maternal clinical dyslipidemia measured during pregnancy and three-month postpartum.**

| **Suboptimal outcomes** | **Retinal arteriolar calibre**  **Per SD increase (8.0 um)** | **Retinal venular calibre**  **Per SD increase (12.2 um)** | | **Fractal dimension arteriole**  **Per SD increase (0.04 df)** | | | **Fractal dimension venule**  **Per SD increase (0.04 df)** | | **Branching angle arteriole**  **Per SD increase (7.9 degree)** | | **Branching angle venule**  **Per SD increase (8.0 degree)** |  |
| --- | --- | --- | --- | --- | --- | --- | --- | --- | --- | --- | --- | --- |
| **Sensitivity analysis 1: Model 2+ fasting glucose (n = 154)** | | | | | | | | | | | |  |
| **Total cholesterol** |  |  | |  | |  | |  | |  | |  |
| Optimal | Ref | Ref | | Ref | | Ref | | Ref | | Ref | |  |
| Ever suboptimal | 1.29(0.87, 1.90) | 1.50(0.99, 2.28) | | 0.78(0.52, 1.16) | | 0.97(0.66, 1.43) | | **1.51(1.00, 2.28)** | | 1.17(0.77, 1.78) | |  |
| Persistently suboptimal | 1.26(0.76, 2.08) | 1.63(0.94, 2.84) | | 0.93(0.54, 1.59) | | 1.01 (0.59, 1.73) | | **2.22(1.23, 4.01)** | | 1.26(0.71, 2.22) | |  |
| **LDL cholesterol** |  |  | |  | |  | |  | |  | |  |
| Optimal | Ref | Ref | | Ref | | Ref | | Ref | | Ref | |  |
| Ever suboptimal | 1.20 (0.82, 1.75) | 1.26(0.84, 1.88) | | 0.94(0.64, 1.39) | | 1.22(0.83, 1.81) | | 1.08(0.72, 1.60) | | 1.31(0.86, 1.99) | |  |
| Persistently suboptimal | 1.36(0.72, 2.58) | **2.19(1.08, 4.44)** | | 0.99(0.51, 1.89) | | 1.22(0.66, 2.27) | | **3.17(1.47, 6.84)** | | 1.00(0.53, 1.88) | |  |
| **Sensitivity analysis 2: Model 2+ high-sensitivity C-reactive protein (n = 153)** | | | | | | | | | | | | |
| **Total cholesterol** |  | |  | |  | |  | |  | |  | |
| Optimal | Ref | | Ref | | Ref | | Ref | | Ref | | Ref | |
| Ever suboptimal | 1.20(0.79, 1.82) | | 1.30(0.83, 2.06) | | 0.85(0.56, 1.29) | | 0.99(0.67, 1.47) | | 1.41(0.93, 2.14) | | 1.29(0.84, 1.99) | |
| Persistently suboptimal | 1.01(0.57, 1.78) | | 1.19(0.64, 2.22) | | 0.97(0.55, 1.71) | | 0.99(0.56, 1.74) | | **2.00(1.12, 3.59)** | | 1.23(0.69, 2.16) | |
| **LDL cholesterol** |  | |  | |  | |  | |  | |  | |
| Normal | Ref | | Ref | | Ref | | Ref | | Ref | | Ref | |
| Ever suboptimal | 1.19(0.80, 1.77) | | 1.20(0.78, 1.86) | | 1.02(0.68, 1.52) | | 1.25(0.84, 1.86) | | 1.00(0.67, 1.51) | | 1.38(0.91, 2.10) | |
| Persistently suboptimal | 1.07(0.53, 2.17) | | 1.67(0.76, 3.70) | | 1.08(0.55, 2.14) | | 1.20(0.63, 2.30) | | 2.88(1.34, 6.21) | | 0.98(0.51, 1.87) | |
| **Sensitivity analysis 3: Model 2+ corresponding lipids (n = 156)** | | | | | | | | | | | | |
| **Total cholesterol** |  | |  | |  | |  | |  | |  | |
| Optimal | Ref | | Ref | | Ref | | Ref | | Ref | | Ref | |
| Ever suboptimal | 1.19(0.73, 1.93) | | 1.44(0.83, 2.49) | | 0.55(0.32, 0.95) | | 0.88(0.55, 1.42) | | **1.90(1.12, 3.21)** | | 1.65(0.96, 2.86) | |
| Persistently suboptimal | 1.08(0.53, 2.21) | | 1.83(0.81, 4.11) | | 0.60(0.27, 1.30) | | 0.71(0.33, 1.53) | | **3.18(1.39, 7.25)** | | 1.89(0.83, 4.31) | |
| **LDL cholesterol** |  | |  | |  | |  | |  | |  | |
| Optimal | Ref | | Ref | | Ref | | Ref | | Ref | | Ref | |
| Ever suboptimal | 1.30(0.76, 2.22) | | 1.32(0.74, 2.35) | | 0.66(0.37, 1.15) | | 1.52(0.90, 2.55) | | 1.10(0.65, 1.86) | | 1.91(1.05, 3.48) | |
| Persistently suboptimal | 1.47(0.61, 3.51) | | **3.54(1.23, 10.15)** | | 0.52(0.20, 1.33) | | 1.34(0.56, 3.18) | | **3.85 (1.36, 10.90)** | | 2.03 (0.77, 5.31) | |
| **Sensitivity analysis 4: Model 2+ obstetric history of GDM (n = 156)** | | | | | | | | | | | | |
| **Total cholesterol** |  | |  | |  | |  | |  | |  | |
| Optimal | Ref | | Ref | | Ref | | Ref | | Ref | | Ref | |
| Ever suboptimal | 1.27(0.85, 1.90) | | 1.48(0.98, 2.24) | | 0.71(0.46, 1.08) | | 0.95(0.65, 1.40) | | 1.52(1.00, 2.31) | | 1.14(0.76, 1.73) | |
| Persistently suboptimal | 1.34(0.79, 2.28) | | 1.74(0.98, 3.09) | | 0.78(0.44, 1.37) | | 0.98(0.57, 1.70) | | **2.22(1.24, 3.99)** | | 1.12(0.64, 1.93) | |
| **LDL cholesterol** |  | |  | |  | |  | |  | |  | |
| Optimal | Ref | | Ref | | Ref | | Ref | | Ref | | Ref | |
| Ever suboptimal | 1.20(0.81, 1.77) | | 1.24(0.83, 1.85) | | 0.86(0.57, 1.29) | | 1.18(0.80, 1.74) | | 1.08(0.72, 1.62) | | 1.28(0.85, 1.93) | |
| Persistently suboptimal | 1.40(0.73, 2.70) | | **2.23(1.10, 4.54)** | | 0.88 (0.45, 1.72) | | 1.20(0.65, 2.24) | | **3.21(1.49, 6.90)** | | 0.93(0.50, 1.72) | |

Model 2, adjusted for age, ethnicity, education, parity, family history of diabetes, pre-pregnancy smoking status and body mass index

| Total cholesterol (mid-pregnancy) | Coefficient | Std. err. | t | P>\|t\| | [95% conf. interval] | |
| --- | --- | --- | --- | --- | --- | --- |
| Retinal venular calibre | 1.05 | 0.86 | 1.22 | 0.22 | -0.65 | 2.75 |
| Pre-pregnancy BMI | -0.04 | 0.02 | -1.80 | 0.07 | -0.09 | 0.00 |
| c.Retinal venular calibre#c.Pre-pregnancy BMI | -.0038229 | .0225667 | -0.17 | 0.866 | -.0483797 | .0407339 |
| Maternal age | .004384 | .0275706 | 0.16 | 0.874 | -.0500526 | .0588206 |
| c.Retinal venular calibre # c.Maternal age | -.0298941 | .0235213 | -1.27 | 0.206 | -.0763357 | .0165475 |
| ethnic_group |  |  |  |  |  |  |
| 2 | -.106591 | .2659801 | -0.40 | 0.689 | -.6317542 | .4185722 |
| 3 | -.6722196 | .2784387 | -2.41 | 0.017 | -1.221982 | -.1224576 |
| 4 | -.274576 | .4477904 | -0.61 | 0.541 | -1.158714 | .6095617 |
| ethnic_group#c.Retinal venular calibre |  |  |  |  |  |  |
| 2 | .0653642 | .283247 | 0.23 | 0.818 | -.4938916 | .62462 |
| 3 | .0681942 | .2620099 | 0.26 | 0.795 | -.4491302 | .5855186 |
| 4 | .33432 | .5990089 | 0.56 | 0.578 | -.8483905 | 1.517031 |
| Family diabetes history | -.2255857 | .1997099 | -1.13 | 0.260 | -.619902 | .1687306 |
| Maternal education | -.0759098 | .2090227 | -0.36 | 0.717 | -.4886137 | .3367942 |
| Parity | -.0590654 | .1745149 | -0.34 | 0.735 | -.4036356 | .2855048 |
| smoking | -.1763278 | .3036897 | -0.58 | 0.562 | -.7759466 | .423291 |

*Supplementary Note 1: Testing interaction effect in the associations of pre-conceptional retinal vascular parameters and maternal lipids at two time points*

| Total cholesterol (3-month postpartum) | Coefficient | Std. err. | t | P>\|t\| | [95% conf. interval] | |
| --- | --- | --- | --- | --- | --- | --- |
| Retinal venular calibre | -1.42 | 0.87 | -1.63 | 0.11 | -3.14 | 0.30 |
| Pre-pregnancy BMI | 0.01 | 0.02 | 0.27 | 0.79 | -0.04 | 0.05 |
| c.Retinal venular calibre#c.Pre-pregnancy BMI | .0198543 | .0236771 | 0.84 | 0.403 | -.0269656 | .0666742 |
| Maternal age | .021447 | .0288165 | 0.74 | 0.458 | -.0355357 | .0784297 |
| c.Maternal age | .0371237 | .0243927 | 1.52 | 0.130 | -.0111113 | .0853587 |
| ethnic_group |  |  |  |  |  |  |
| 2 | -.0616381 | .2968576 | -0.21 | 0.836 | -.6486535 | .5253774 |
| 3 | -.3359944 | .3016469 | -1.11 | 0.267 | -.9324804 | .2604917 |
| 4 | .8410754 | 1.539635 | 0.55 | 0.586 | -2.203447 | 3.885598 |
| ethnic_group#c.Retinal venular calibre |  |  |  |  |  |  |
| 2 | .1326218 | .3001329 | 0.44 | 0.659 | -.4608703 | .7261139 |
| 3 | .1731089 | .2628072 | 0.66 | 0.511 | -.3465743 | .6927922 |
| 4 | -.630694 | 2.136332 | -0.30 | 0.768 | -4.855143 | 3.593755 |
| Family diabetes history | -.3560719 | .2138458 | -1.67 | 0.098 | -.7789373 | .0667934 |
| Maternal education | .3976813 | .2248454 | 1.77 | 0.079 | -.0469351 | .8422977 |
| Parity | -.4757172 | .1823472 | -2.61 | 0.010 | -.8362963 | -.115138 |
| smoking | .1649174 | .3138868 | 0.53 | 0.600 | -.4557721 | .785607 |
| _cons | -.8869241 | .9606923 | -0.92 | 0.358 | -2.786627 | 1.012779 |

| HDL-cholesterol (mid-pregnancy) | Coefficient | Std. err. | t | P>\|t\| | [95% conf. interval] | |
| --- | --- | --- | --- | --- | --- | --- |
| Arteriolar fractal dimension | -0.86 | 0.82 | -1.05 | 0.30 | -2.48 | 0.76 |
| Pre-pregnancy BMI | -0.04 | 0.02 | -2.03 | 0.04 | -0.08 | -0.00 |
| c.Arteriolar fractal dimension#c.Pre-pregnancy BMI | .0192519 | .0204613 | 0.94 | 0.348 | -.0211479 | .0596517 |
| Maternal age | -.0027802 | .0255297 | -0.11 | 0.913 | -.0531872 | .0476268 |
| c.Maternal age | .0086897 | .0226665 | 0.38 | 0.702 | -.0360639 | .0534434 |
| ethnic_group |  |  |  |  |  |  |
| 2 | -.3755607 | .2420879 | -1.55 | 0.123 | -.85355 | .1024287 |
| 3 | -1.177625 | .2572319 | -4.58 | 0.000 | -1.685516 | -.6697349 |
| 4 | -.3668834 | .4159287 | -0.88 | 0.379 | -1.188112 | .4543451 |
| ethnic_group#c.Arteriolar fractal dimension |  |  |  |  |  |  |
| 2 | .0443142 | .2085432 | 0.21 | 0.832 | -.367443 | .4560714 |
| 3 | .1189315 | .4347672 | 0.27 | 0.785 | -.7394927 | .9773557 |
| 4 | -.1728786 | .3102464 | -0.56 | 0.578 | -.7854432 | .439686 |
| Family diabetes history | .0061419 | .18298 | 0.03 | 0.973 | -.3551421 | .367426 |
| Maternal education | -.1020364 | .1902691 | -0.54 | 0.592 | -.4777123 | .2736396 |
| Parity | -.2584181 | .1603379 | -1.61 | 0.109 | -.5749965 | .0581603 |
| smoking | -.1872805 | .2750173 | -0.68 | 0.497 | -.7302871 | .3557262 |
| _cons | 1.319997 | .8886805 | 1.49 | 0.139 | -.4346543 | 3.074648 |

| HDL-cholesterol (3-month postpartum) | Coefficient | Std. err. | t | P>\|t\| | [95% conf. interval] | |
| --- | --- | --- | --- | --- | --- | --- |
| Arteriolar fractal dimension | -0.37 | 0.76 | -0.49 | 0.63 | -1.87 | 1.13 |
| Pre-pregnancy BMI | -0.09 | 0.02 | -4.42 | 0.00 | -0.12 | -0.05 |
| c.Arteriolar fractal dimension#c.Pre-pregnancy BMI | .0312219 | .0196455 | 1.59 | 0.114 | -.0076258 | .0700695 |
| Maternal age | .0167338 | .0241402 | 0.69 | 0.489 | -.0310018 | .0644694 |
| c.Maternal age | -.0109723 | .0210108 | -0.52 | 0.602 | -.0525197 | .0305751 |
| ethnic_group |  |  |  |  |  |  |
| 2 | .1032587 | .2362196 | 0.44 | 0.663 | -.3638493 | .5703667 |
| 3 | -.6545445 | .247155 | -2.65 | 0.009 | -1.143277 | -.1658126 |
| 4 | .5986972 | .5359192 | 1.12 | 0.266 | -.4610461 | 1.658441 |
| ethnic_group#c.Arteriolar fractal dimension |  |  |  |  |  |  |
| 2 | -.2301749 | .1880081 | -1.22 | 0.223 | -.601948 | .1415983 |
| 3 | -.8048247 | .3892523 | -2.07 | 0.041 | -1.574544 | -.035105 |
| 4 | -.7012102 | .3348259 | -2.09 | 0.038 | -1.363306 | -.039115 |
| Family diabetes history | -.1274584 | .1752318 | -0.73 | 0.468 | -.4739673 | .2190504 |
| Maternal education | .2635655 | .1837977 | 1.43 | 0.154 | -.0998818 | .6270127 |
| Parity | -.0876524 | .1543396 | -0.57 | 0.571 | -.3928483 | .2175435 |
| smoking | .1414802 | .2612892 | 0.54 | 0.589 | -.3752011 | .6581616 |
| _cons | 1.321273 | .8264126 | 1.60 | 0.112 | -.3129013 | 2.955447 |

| Total cholesterol (mid-pregnancy) | Coefficient | Std. err. | t | P>\|t\| | [95% conf. interval] | |
| --- | --- | --- | --- | --- | --- | --- |
| Arteriolar branching angle | 1.14 | 0.96 | 1.20 | 0.23 | -0.74 | 3.03 |
| Pre-pregnancy BMI | -0.04 | 0.02 | -1.80 | 0.07 | -0.08 | 0.00 |
| c.Arteriolar branching angle#c.Pre-pregnancy BMI | -.0239927 | .0202017 | -1.19 | 0.237 | -.0638798 | .0158944 |
| Maternal age | .0000904 | .0267495 | 0.00 | 0.997 | -.0527251 | .0529059 |
| c.Arteriolar branching angle#c.Maternal age | -.0140537 | .026625 | -0.53 | 0.598 | -.0666233 | .038516 |
| ethnic_group |  |  |  |  |  |  |
| 2 | -.1388975 | .2582327 | -0.54 | 0.591 | -.648764 | .3709691 |
| 3 | -.6924819 | .2768002 | -2.50 | 0.013 | -1.239009 | -.1459551 |
| 4 | -.288733 | .4429466 | -0.65 | 0.515 | -1.163307 | .5858409 |
| ethnic_group#c.Arteriolar branching angle |  |  |  |  |  |  |
| 2 | .0877822 | .2907342 | 0.30 | 0.763 | -.4862566 | .661821 |
| 3 | -.1558012 | .2928476 | -0.53 | 0.595 | -.7340128 | .4224105 |
| 4 | -.1694577 | .4042444 | -0.42 | 0.676 | -.9676163 | .6287008 |
| Family diabetes history | -.2341958 | .199968 | -1.17 | 0.243 | -.6290216 | .1606301 |
| Maternal education | -.1353626 | .2047506 | -0.66 | 0.509 | -.5396315 | .2689063 |
| Parity | -.0124991 | .1705505 | -0.07 | 0.942 | -.3492418 | .3242437 |
| smoking | -.2054143 | .2960843 | -0.69 | 0.489 | -.7900166 | .379188 |
| _cons | 1.069616 | .9187182 | 1.16 | 0.246 | -.7443428 | 2.883575 |

| Total cholesterol (3-month postpartum) | Coefficient | Std. err. | t | P>\|t\| | [95% conf. interval] | |
| --- | --- | --- | --- | --- | --- | --- |
| Arteriolar branching angle | 1.01 | 1.08 | 0.94 | 0.35 | -1.12 | 3.15 |
| Pre-pregnancy BMI | 0.01 | 0.02 | 0.63 | 0.53 | -0.03 | 0.06 |
| c.Arteriolar branching angle#c.Pre-pregnancy BMI | -.0267188 | .0217222 | -1.23 | 0.221 | -.069673 | .0162354 |
| Maternal age | .0334685 | .0284652 | 1.18 | 0.242 | -.0228195 | .0897566 |
|  |  |  |  |  |  |  |
| c.Arteriolar branching angle#c.Maternal age | -.0046821 | .0309748 | -0.15 | 0.880 | -.0659326 | .0565684 |
| ethnic_group |  |  |  |  |  |  |
| 2 | .1387614 | .2787759 | 0.50 | 0.619 | -.4124988 | .6900216 |
| 3 | -.3204559 | .3049992 | -1.05 | 0.295 | -.9235707 | .282659 |
| 4 | .5294715 | .6851425 | 0.77 | 0.441 | -.8253507 | 1.884294 |
| ethnic_group#c.Arteriolar branching angle |  |  |  |  |  |  |
| 2 | -.2796871 | .3210499 | -0.87 | 0.385 | -.9145412 | .355167 |
| 3 | -.4007247 | .3411068 | -1.17 | 0.242 | -1.07524 | .2737906 |
| 4 | -.0031206 | .6176829 | -0.01 | 0.996 | -1.224546 | 1.218305 |
| Family diabetes history | -.4172733 | .2122669 | -1.97 | 0.051 | -.8370164 | .0024699 |
| Maternal education | .3693288 | .2219984 | 1.66 | 0.098 | -.0696577 | .8083154 |
| Parity | -.4244061 | .1822831 | -2.33 | 0.021 | -.7848583 | -.0639538 |
| smoking | .1558976 | .317428 | 0.49 | 0.624 | -.4717945 | .7835897 |
| _cons | -1.422842 | .9577719 | -1.49 | 0.140 | -3.31677 | .4710865 |

| LDL-cholesterol (mid-pregnancy) | Coefficient | Std. err. | t | P>\|t\| | [95% conf. interval] | |
| --- | --- | --- | --- | --- | --- | --- |
| Arteriolar branching angle | 0.66 | 0.96 | 0.69 | 0.49 | -1.23 | 2.55 |
| Pre-pregnancy BMI | -0.04 | 0.02 | -1.79 | 0.08 | -0.08 | 0.00 |
| c.Arteriolar branching angle#c.Pre-pregnancy BMI | -.0135432 | .0202446 | -0.67 | 0.504 | -.0535168 | .0264305 |
| Maternal age | -.0020019 | .0268165 | -0.07 | 0.941 | -.054952 | .0509482 |
| c.Arteriolar branching angle#c.Maternal age | -.0045713 | .026687 | -0.17 | 0.864 | -.0572658 | .0481232 |
| ethnic_group |  |  |  |  |  |  |
| 2 | .0077681 | .2588746 | 0.03 | 0.976 | -.5033888 | .518925 |
| 3 | -.2366687 | .2774361 | -0.85 | 0.395 | -.7844759 | .3111385 |
| 4 | -.3011466 | .443989 | -0.68 | 0.499 | -1.177818 | .5755251 |
| ethnic_group#c.Arteriolar branching angle |  |  |  |  |  |  |
| 2 | -.0075264 | .2913505 | -0.03 | 0.979 | -.5828079 | .5677552 |
| 3 | -.2357663 | .2934719 | -0.80 | 0.423 | -.8152367 | .343704 |
| 4 | -.0962359 | .4051091 | -0.24 | 0.813 | -.8961378 | .703666 |
| Family diabetes history | -.2392291 | .2004661 | -1.19 | 0.234 | -.6350563 | .1565982 |
| Maternal education | -.158482 | .2053567 | -0.77 | 0.441 | -.5639658 | .2470018 |
| Parity | .0635282 | .1718442 | 0.37 | 0.712 | -.2757841 | .4028404 |
| smoking | -.1652296 | .29676 | -0.56 | 0.578 | -.7511925 | .4207333 |
| _cons | 1.086006 | .9208961 | 1.18 | 0.240 | -.7323348 | 2.904348 |

| LDL-cholesterol (3-month postpartum) | Coefficient | Std. err. | t | P>\|t\| | [95% conf. interval] | |
| --- | --- | --- | --- | --- | --- | --- |
| Arteriolar branching angle | 0.68 | 1.10 | 0.62 | 0.54 | -1.49 | 2.85 |
| Pre-pregnancy BMI | 0.04 | 0.02 | 1.53 | 0.13 | -0.01 | 0.08 |
| c.Arteriolar branching angle#c.Pre-pregnancy BMI | -.0097238 | .022148 | -0.44 | 0.661 | -.0535199 | .0340724 |
| Maternal age | .0220131 | .0290232 | 0.76 | 0.449 | -.0353783 | .0794045 |
|  |  |  |  |  |  |  |
| c.Arteriolar branching angle#c.Maternal age | -.0070556 | .0315819 | -0.22 | 0.824 | -.0695067 | .0553955 |
| ethnic_group |  |  |  |  |  |  |
| 2 | .1164955 | .2842403 | 0.41 | 0.683 | -.4455701 | .6785611 |
| 3 | -.1457888 | .3109775 | -0.47 | 0.640 | -.7607255 | .4691479 |
| 4 | .4145059 | .6985722 | 0.59 | 0.554 | -.9668726 | 1.795884 |
| ethnic_group#c.Arteriolar branching angle |  |  |  |  |  |  |
| 2 | -.378648 | .3273429 | -1.16 | 0.249 | -1.025946 | .2686501 |
| 3 | -.2888212 | .347793 | -0.83 | 0.408 | -.9765579 | .3989155 |
| 4 | -.0711685 | .6297903 | -0.11 | 0.910 | -1.316535 | 1.174198 |
| Family diabetes history | -.4021096 | .2164276 | -1.86 | 0.065 | -.8300802 | .025861 |
| Maternal education | .3334032 | .2263498 | 1.47 | 0.143 | -.114188 | .7809944 |
| Parity | -.3543549 | .1858561 | -1.91 | 0.059 | -.7218724 | .0131627 |
| smoking | .0809604 | .32365 | 0.25 | 0.803 | -.5590352 | .7209561 |
| _cons | -1.562122 | .9765455 | -1.60 | 0.112 | -3.493174 | .3689292 |

Supplementary Note 2: Testing interaction effect in the associations of pre-conceptional retinal vascular parameters and maternal lipids in linear mixed models

| Total cholesterol from mid-pregancny to postpartum | Coefficient | Std. err. | z | P>\|z\| | [95% conf. interval] | |
| --- | --- | --- | --- | --- | --- | --- |
| Arteriolar branching angle | 1.10 | 0.84 | 1.31 | 0.19 | -0.55 | 2.75 |
| Pre-pregnancy BMI | -0.02 | 0.02 | -1.07 | 0.28 | -0.06 | 0.02 |
| c.Arteriolar branching angle#c.Pre-pregnancy BMI | -.024037 | .0178532 | -1.35 | 0.178 | -.0590286 | .0109545 |
| Maternal age | .0245223 | .0234962 | 1.04 | 0.297 | -.0215293 | .070574 |
| c.Arteriolar branching angle#c.Maternal age | -.0127413 | .023451 | -0.54 | 0.587 | -.0587045 | .0332218 |
| ethnic_group |  |  |  |  |  |  |
| 2 | -.1717563 | .2271223 | -0.76 | 0.450 | -.6169079 | .2733952 |
| 3 | -.6135121 | .2454944 | -2.50 | 0.012 | -1.094672 | -.1323519 |
| 4 | -.2639276 | .409195 | -0.64 | 0.519 | -1.065935 | .53808 |
| ethnic_group#c.Arteriolar branching angle |  |  |  |  |  |  |
| 2 | -.035553 | .258552 | -0.14 | 0.891 | -.5423057 | .4711996 |
| 3 | -.2456914 | .2623855 | -0.94 | 0.349 | -.7599576 | .2685748 |
| 4 | -.1243085 | .3731738 | -0.33 | 0.739 | -.8557158 | .6070987 |
| Family diabetes history | -.1649148 | .1718342 | -0.96 | 0.337 | -.5017036 | .171874 |
| Maternal education | -.0196382 | .1798414 | -0.11 | 0.913 | -.3721209 | .3328444 |
| Parity | -.136382 | .1488937 | -0.92 | 0.360 | -.4282082 | .1554443 |
| smoking | .0642845 | .2555725 | 0.25 | 0.801 | -.4366283 | .5651974 |
| _cons | -.1242456 | .8056895 | -0.15 | 0.877 | -1.703368 | 1.454877 |

| Triglyceride from mid-pregancny to postpartum | Coefficient | Std. err. | z | P>\|z\| | [95% conf. interval] | |
| --- | --- | --- | --- | --- | --- | --- |
| Arteriolar fractal dimension | -0.41 | 0.64 | -0.64 | 0.52 | -1.66 | 0.84 |
| Pre-pregnancy BMI | 0.06 | 0.02 | 3.96 | 0.00 | 0.03 | 0.10 |
| c.Arteriolar fractal dimension#c.Pre-pregnancy BMI | -.0016072 | .0162539 | -0.10 | 0.921 | -.0334642 | .0302498 |
| Maternal age | .0184428 | .0200808 | 0.92 | 0.358 | -.0209148 | .0578004 |
| c.Maternal age | .0154434 | .0176569 | 0.87 | 0.382 | -.0191636 | .0500504 |
| ethnic_group |  |  |  |  |  |  |
| 2 | -.2207982 | .192096 | -1.15 | 0.250 | -.5972994 | .155703 |
| 3 | -.1077206 | .2044569 | -0.53 | 0.598 | -.5084489 | .2930076 |
| 4 | .2796359 | .3557232 | 0.79 | 0.432 | -.4175688 | .9768406 |
| ethnic_group#c.Arteriolar fractal dimension |  |  |  |  |  |  |
| 2 | .2463978 | .16228 | 1.52 | 0.129 | -.0716651 | .5644607 |
| 3 | .4564578 | .3378271 | 1.35 | 0.177 | -.2056712 | 1.118587 |
| 4 | -.111369 | .254821 | -0.44 | 0.662 | -.6108091 | .388071 |
| Family diabetes history | .0547302 | .141667 | 0.39 | 0.699 | -.2229319 | .3323924 |
| Maternal education | -.086275 | .1505243 | -0.57 | 0.567 | -.3812973 | .2087473 |
| Parity | -.0755906 | .1256364 | -0.60 | 0.547 | -.3218334 | .1706523 |
| smoking | .3127121 | .2124865 | 1.47 | 0.141 | -.1037537 | .7291778 |
| _cons | -1.95757 | .6962632 | -2.81 | 0.005 | -3.32222 | -.5929189 |

| HDL-cholesterol from mid-pregancny to postpartum | Coefficient | Std. err. | z | P>\|z\| | [95% conf. interval] | |
| --- | --- | --- | --- | --- | --- | --- |
| Arteriolar fractal dimension | -0.55 | 0.63 | -0.86 | 0.39 | -1.79 | 0.69 |
| Pre-pregnancy BMI | -0.06 | 0.02 | -3.99 | 0.00 | -0.10 | -0.03 |
| c.Arteriolar fractal dimension#c.Pre-pregnancy BMI | .0237094 | .0160558 | 1.48 | 0.140 | -.0077594 | .0551782 |
| Maternal age | .0080168 | .0196608 | 0.41 | 0.683 | -.0305176 | .0465513 |
| c.Maternal age | -.0026977 | .0174873 | -0.15 | 0.877 | -.0369722 | .0315769 |
| ethnic_group |  |  |  |  |  |  |
| 2 | -.2010939 | .1894535 | -1.06 | 0.288 | -.572416 | .1702281 |
| 3 | -.9479816 | .2021376 | -4.69 | 0.000 | -1.344164 | -.5517993 |
| 4 | -.1344832 | .3453746 | -0.39 | 0.697 | -.8114049 | .5424386 |
| ethnic_group#c.Arteriolar fractal dimension |  |  |  |  |  |  |
| 2 | -.0752792 | .1605157 | -0.47 | 0.639 | -.3898842 | .2393258 |
| 3 | -.3295516 | .3354661 | -0.98 | 0.326 | -.9870531 | .3279499 |
| 4 | -.3369575 | .2500139 | -1.35 | 0.178 | -.8269757 | .1530607 |
| Family diabetes history | -.0290965 | .1398634 | -0.21 | 0.835 | -.3032237 | .2450308 |
| Maternal education | .0225982 | .1443294 | 0.16 | 0.876 | -.2602821 | .3054786 |
| Parity | -.1468065 | .1241912 | -1.18 | 0.237 | -.3902169 | .0966038 |
| _cons | 1.337917 | .6873235 | 1.95 | 0.052 | -.0092125 | 2.685046 |

| LDL-cholesterol from mid-pregancny to postpartum | Coefficient | Std. err. | z | P>\|z\| | [95% conf. interval] | |
| --- | --- | --- | --- | --- | --- | --- |
| Retinal venular calibre | -0.02 | 0.75 | -0.02 | 0.98 | -1.49 | 1.46 |
| Pre-pregnancy BMI | -0.02 | 0.02 | -0.97 | 0.33 | -0.06 | 0.02 |
| c.Retinal venular calibre#c.Pre-pregnancy BMI | .0023201 | .0198294 | 0.12 | 0.907 | -.0365447 | .0411849 |
| Maternal age | .0143289 | .0241461 | 0.59 | 0.553 | -.0329967 | .0616545 |
| c.Maternal age | .0014925 | .0206122 | 0.07 | 0.942 | -.0389066 | .0418916 |
| ethnic_group |  |  |  |  |  |  |
| 2 | -.1146104 | .2352882 | -0.49 | 0.626 | -.5757668 | .346546 |
| 3 | -.2526763 | .2455893 | -1.03 | 0.304 | -.7340225 | .2286699 |
| 4 | -.3350433 | .4123968 | -0.81 | 0.417 | -1.143326 | .4732396 |
| ethnic_group#c.Retinal venular calibre |  |  |  |  |  |  |
| 2 | .0934292 | .2493619 | 0.37 | 0.708 | -.395311 | .5821695 |
| 3 | .203171 | .2284231 | 0.89 | 0.374 | -.2445301 | .650872 |
| 4 | .5087873 | .5541328 | 0.92 | 0.359 | -.577293 | 1.594868 |
| Family diabetes history | -.1432217 | .171629 | -0.83 | 0.404 | -.4796084 | .193165 |
| Maternal education | .0268954 | .1831409 | 0.15 | 0.883 | -.3320542 | .3858451 |
| Parity | -.1149886 | .1523459 | -0.75 | 0.450 | -.413581 | .1836037 |
| smoking | .017535 | .2598343 | 0.07 | 0.946 | -.491731 | .5268009 |
| _cons | .093185 | .8186752 | 0.11 | 0.909 | -1.511389 | 1.697759 |

| LDL-cholesterol from mid-pregancny to postpartum | Coefficient | Std. err. | z | P>\|z\| | [95% conf. interval] | |
| --- | --- | --- | --- | --- | --- | --- |
| Arteriolar branching angle | 0.68 | 0.84 | 0.80 | 0.42 | -0.97 | 2.32 |
| Pre-pregnancy BMI | -0.01 | 0.02 | -0.63 | 0.53 | -0.05 | 0.03 |
| c.Arteriolar branching angle#c.Pre-pregnancy BMI | -.0115441 | .0178107 | -0.65 | 0.517 | -.0464524 | .0233641 |
| Maternal age | .0153895 | .0234484 | 0.66 | 0.512 | -.0305686 | .0613475 |
| c.Arteriolar branching angle#c.Maternal age | -.007401 | .0233907 | -0.32 | 0.752 | -.0532459 | .0384439 |
| ethnic_group |  |  |  |  |  |  |
| 2 | -.088948 | .2266489 | -0.39 | 0.695 | -.5331718 | .3552758 |
| 3 | -.2663129 | .2449192 | -1.09 | 0.277 | -.7463457 | .2137199 |
| 4 | -.2996285 | .4079133 | -0.73 | 0.463 | -1.099124 | .4998668 |
| ethnic_group#c.Arteriolar branching angle |  |  |  |  |  |  |
| 2 | -.1384906 | .2579088 | -0.54 | 0.591 | -.6439826 | .3670015 |
| 3 | -.292083 | .261692 | -1.12 | 0.264 | -.8049899 | .2208238 |
| 4 | -.0768182 | .371938 | -0.21 | 0.836 | -.8058033 | .6521668 |
| Family diabetes history | -.175161 | .1714888 | -1.02 | 0.307 | -.5112729 | .1609509 |
| Maternal education | -.035507 | .1795171 | -0.20 | 0.843 | -.3873542 | .3163401 |
| Parity | -.0513644 | .1491968 | -0.34 | 0.731 | -.3437849 | .241056 |
| smoking | -.0090854 | .2549993 | -0.04 | 0.972 | -.5088748 | .4907041 |
| _cons | -.0758636 | .8040225 | -0.09 | 0.925 | -1.651719 | 1.499992 |

Supplementary Note 3: Testing interaction effect in the associations of pre-conceptional retinal vascular parameters and clinical maternal lipid categories

| B_LDL | RRR | Std. err. | z | P>\|z\| | [95% conf. interval] | |
| --- | --- | --- | --- | --- | --- | --- |
| 0 | (base outcome) |  |  |  |  |  |
| 1 |  |  |  |  |  |  |
| Retinal venular calibre | .0937873 | .2024202 | -1.10 | 0.273 | .0013646 | 6.445915 |
| Pre-pregnancy BMI | 1.11982 | .0636075 | 1.99 | 0.046 | 1.001841 | 1.251693 |
| c.Retinal venular calibre#c.Pre-pregnancy BMI | 1.051264 | .0642141 | 0.82 | 0.413 | .9326485 | 1.184965 |
| Maternal age | .9838002 | .0680456 | -0.24 | 0.813 | .8590782 | 1.12663 |
| c.Maternal age | 1.045784 | .063201 | 0.74 | 0.459 | .928967 | 1.17729 |
| ethnic_group |  |  |  |  |  |  |
| 2 | 1.114326 | .8329402 | 0.14 | 0.885 | .2574857 | 4.82249 |
| 3 | .7075931 | .5742964 | -0.43 | 0.670 | .1441889 | 3.472445 |
| 4 | 1.86e+17 | 6.66e+20 | 0.01 | 0.991 | 0 | . |
| ethnic_group#c.Retinal venular calibre |  |  |  |  |  |  |
| 2 | 1.398057 | 1.000651 | 0.47 | 0.640 | .3437814 | 5.685481 |
| 3 | 1.82323 | 1.484041 | 0.74 | 0.461 | .3698239 | 8.988512 |
| 4 | 3.74e-33 | 2.22e-29 | -0.01 | 0.990 | 0 | . |
| Family diabetes history | .7783704 | .4028676 | -0.48 | 0.628 | .2822428 | 2.146593 |
| Maternal education | 2.012839 | 1.147776 | 1.23 | 0.220 | .65831 | 6.154425 |
| Parity | .6116877 | .2788929 | -1.08 | 0.281 | .2502839 | 1.49495 |
| _cons | .0325429 | .0757672 | -1.47 | 0.141 | .0003394 | 3.120657 |
| 2 |  |  |  |  |  |  |
| Retinal venular calibre | 284.239 | 1275.785 | 1.26 | 0.208 | .0429666 | 1880341 |
| Pre-pregnancy BMI | 1.100412 | .1174278 | 0.90 | 0.370 | .8927324 | 1.356404 |
| c.Retinal venular calibre#c.Pre-pregnancy BMI | .9142729 | .1028678 | -0.80 | 0.426 | .7333384 | 1.139849 |
| Maternal age | .8799964 | .1152624 | -0.98 | 0.329 | .6807537 | 1.137553 |
| c.Maternal age | .9137748 | .1082477 | -0.76 | 0.447 | .7244427 | 1.152589 |
| ethnic_group |  |  |  |  |  |  |
| 2 | .4737857 | .7243707 | -0.49 | 0.625 | .023669 | 9.483824 |
| 3 | .8050804 | 1.094268 | -0.16 | 0.873 | .0560896 | 11.55569 |
| 4 | .0006023 | 14.44539 | -0.00 | 1.000 | 0 | . |
| ethnic_group#c.Retinal venular calibre |  |  |  |  |  |  |
| 2 | .7630821 | 1.114262 | -0.19 | 0.853 | .043615 | 13.35077 |
| 3 | .2027543 | .2229227 | -1.45 | 0.147 | .0235016 | 1.749213 |
| 4 | .000055 | 1.760395 | -0.00 | 1.000 | 0 | . |
| Family diabetes history | .7122562 | .6601364 | -0.37 | 0.714 | .1158036 | 4.380771 |
| Maternal education | 1.748756 | 1.582359 | 0.62 | 0.537 | .2968388 | 10.30238 |
| Parity | .8593988 | .6214178 | -0.21 | 0.834 | .2083072 | 3.545563 |
| _cons | .4329683 | 1.850054 | -0.20 | 0.845 | .0000998 | 1877.594 |

| B_CHO | RRR | Std. err. | z | P>\|z\| | [95% conf. interval] | |
| --- | --- | --- | --- | --- | --- | --- |
| 0 | (base outcome) |  |  |  |  |  |
| 1 |  |  |  |  |  |  |
| Arteriolar branching angle | 1144.182 | 3331.91 | 2.42 | 0.016 | 3.799767 | 344534.8 |
| Pre-pregnancy BMI | 1.093123 | .0651075 | 1.49 | 0.135 | .9726818 | 1.228478 |
| c.Arteriolar branching angle#c.Pre-pregnancy BMI | .8927496 | .0477451 | -2.12 | 0.034 | .8039084 | .9914088 |
| Maternal age | 1.030305 | .0713988 | 0.43 | 0.667 | .8994532 | 1.180192 |
| c.Arteriolar branching angle#c.Maternal age | .8788415 | .0716922 | -1.58 | 0.113 | .7489851 | 1.031212 |
| ethnic_group |  |  |  |  |  |  |
| 2 | 1.982905 | 1.466856 | 0.93 | 0.355 | .4651842 | 8.452381 |
| 3 | 1.040454 | .7730044 | 0.05 | 0.957 | .2425625 | 4.462953 |
| 4 | 3.092567 | 3822.668 | 0.00 | 0.999 | 0 | . |
| ethnic_group#c.Arteriolar branching angle |  |  |  |  |  |  |
| 2 | .5105509 | .4258493 | -0.81 | 0.420 | .0995529 | 2.618329 |
| 3 | .7073363 | .5616302 | -0.44 | 0.663 | .1491989 | 3.353406 |
| 4 | 4.15e+08 | 7.55e+11 | 0.01 | 0.991 | 0 | . |
| Family diabetes history | .589018 | .3160049 | -0.99 | 0.324 | .2058086 | 1.685751 |
| Maternal education | 4.136162 | 2.666957 | 2.20 | 0.028 | 1.16883 | 14.63673 |
| Parity | .3951707 | .1885989 | -1.95 | 0.052 | .1550747 | 1.006998 |
| _cons | .0097145 | .0242265 | -1.86 | 0.063 | .0000732 | 1.288816 |
| 2 |  |  |  |  |  |  |
| Arteriolar branching angle | 20.36258 | 86.72328 | 0.71 | 0.479 | .0048262 | 85913.32 |
| Pre-pregnancy BMI | .9493881 | .0826436 | -0.60 | 0.551 | .8004741 | 1.126005 |
| c.Arteriolar branching angle#c.Pre-pregnancy BMI | .98375 | .0825372 | -0.20 | 0.845 | .8345808 | 1.159581 |
| Maternal age | .9401836 | .102513 | -0.57 | 0.572 | .7592797 | 1.164189 |
| c.Arteriolar branching angle#c.Maternal age | .9464274 | .1128098 | -0.46 | 0.644 | .7492522 | 1.195492 |
| ethnic_group |  |  |  |  |  |  |
| 2 | 1.459672 | 1.228854 | 0.45 | 0.653 | .2803201 | 7.60075 |
| 3 | 4.92e-07 | .0005559 | -0.01 | 0.990 | 0 | . |
| 4 | 2.06e-06 | .0152225 | -0.00 | 0.999 | 0 | . |
| ethnic_group#c.Arteriolar branching angle |  |  |  |  |  |  |
| 2 | .3687391 | .3374968 | -1.09 | 0.276 | .061325 | 2.217178 |
| 3 | .4382481 | 603.0153 | -0.00 | 1.000 | 0 | . |
| 4 | 2.612966 | 23724.26 | 0.00 | 1.000 | 0 | . |
| Family diabetes history | 1.188479 | .8458727 | 0.24 | 0.808 | .2945564 | 4.795288 |
| Maternal education | .7993223 | .5562442 | -0.32 | 0.748 | .2043503 | 3.126574 |
| Parity | 1.034208 | .639497 | 0.05 | 0.957 | .3078024 | 3.474912 |
| _cons | 3.789864 | 13.99637 | 0.36 | 0.718 | .002723 | 5274.738 |

| B_LDL | RRR | Std. err. | z | P>\|z\| | [95% conf. interval] | |
| --- | --- | --- | --- | --- | --- | --- |
| 0 | (base outcome) |  |  |  |  |  |
| 1 |  |  |  |  |  |  |
| Arteriolar branching angle | 28.66067 | 75.37527 | 1.28 | 0.202 | .1654604 | 4964.536 |
| Pre-pregnancy BMI | 1.146899 | .0646583 | 2.43 | 0.015 | 1.026922 | 1.280893 |
| c.Arteriolar branching angle#c.Pre-pregnancy BMI | .986332 | .0508319 | -0.27 | 0.789 | .8915698 | 1.091166 |
| Maternal age | 1.01076 | .0680867 | 0.16 | 0.874 | .8857467 | 1.153418 |
| c.Arteriolar branching angle#c.Maternal age | .908024 | .0692109 | -1.27 | 0.206 | .7820194 | 1.054331 |
| ethnic_group |  |  |  |  |  |  |
| 2 | 1.735663 | 1.12718 | 0.85 | 0.396 | .4860436 | 6.198055 |
| 3 | .7949929 | .6091814 | -0.30 | 0.765 | .177056 | 3.569569 |
| 4 | 1.502583 | 1772.4 | 0.00 | 1.000 | 0 | . |
| ethnic_group#c.Arteriolar branching angle |  |  |  |  |  |  |
| 2 | .3901918 | .3009454 | -1.22 | 0.222 | .0860532 | 1.76925 |
| 3 | 1.154954 | .9902067 | 0.17 | 0.867 | .2151702 | 6.199367 |
| 4 | 7.47e+08 | 1.30e+12 | 0.01 | 0.991 | 0 | . |
| Family diabetes history | .6587271 | .3296993 | -0.83 | 0.404 | .2469852 | 1.756872 |
| Maternal education | 1.975547 | 1.082758 | 1.24 | 0.214 | .6747778 | 5.78381 |
| Parity | .6437746 | .2858881 | -0.99 | 0.321 | .2696071 | 1.537221 |
| _cons | .008725 | .020359 | -2.03 | 0.042 | .0000901 | .8452112 |
| 2 |  |  |  |  |  |  |
| Arteriolar branching angle | .4556673 | 2.372121 | -0.15 | 0.880 | .0000169 | 12298.39 |
| Pre-pregnancy BMI | 1.035618 | .1449729 | 0.25 | 0.803 | .7871226 | 1.362563 |
| c.Arteriolar branching angle#c.Pre-pregnancy BMI | 1.048933 | .1212205 | 0.41 | 0.679 | .8363313 | 1.315581 |
| Arteriolar branching angle | (omitted) |  |  |  |  |  |
| Maternal age | .9061008 | .1418484 | -0.63 | 0.529 | .6666877 | 1.231489 |
| c.Arteriolar branching angle#c.Maternal age | 1.033949 | .1599438 | 0.22 | 0.829 | .7635281 | 1.400146 |
| ethnic_group |  |  |  |  |  |  |
| 2 | .4542028 | .8420333 | -0.43 | 0.670 | .0120014 | 17.18967 |
| 3 | 1.634581 | 2.215729 | 0.36 | 0.717 | .114703 | 23.29368 |
| 4 | 4.06e-06 | .035892 | -0.00 | 0.999 | 0 | . |
| ethnic_group#c.Arteriolar branching angle |  |  |  |  |  |  |
| 2 | .7927603 | 1.317238 | -0.14 | 0.889 | .0305351 | 20.58188 |
| 3 | .3657969 | .5622317 | -0.65 | 0.513 | .0179862 | 7.439455 |
| 4 | 1.95444 | 21597.5 | 0.00 | 1.000 | 0 | . |
| Family diabetes history | .5150496 | .5270577 | -0.65 | 0.517 | .069311 | 3.827331 |
| Maternal education | 1.375377 | 1.268986 | 0.35 | 0.730 | .2254572 | 8.390341 |
| Parity | 1.08659 | .8335163 | 0.11 | 0.914 | .2416099 | 4.886714 |
| _cons | .5584716 | 2.863998 | -0.11 | 0.910 | .0000241 | 12947.92 |
